# Arctigenin derivative A‐1 ameliorates motor dysfunction and pathological manifestations in SOD1^G93A^ transgenic mice via the AMPK/SIRT1/PGC‐1α and AMPK/SIRT1/IL‐1β/NF‐κB pathways

**DOI:** 10.1111/cns.14692

**Published:** 2024-06-13

**Authors:** Bocheng Xiong, Chao Yang, Xiao Yang, Song Luo, Shangming Li, Chongyang Chen, Kaiwu He, Lulin Nie, Peimao Li, Shupeng Li, Haiyan Huang, Jianjun Liu, Zaijun Zhang, Yongmei Xie, Liangyu Zou, Xifei Yang

**Affiliations:** ^1^ Shenzhen Key Laboratory of Modern Toxicology, Shenzhen Medical Key Discipline of Health Toxicology (2020‐2024) Shenzhen Center for Disease Control and Prevention Shenzhen China; ^2^ Department of Neurology The First Affiliated Hospital of Bengbu Medical University Bengbu China; ^3^ Department of Neurology Shenzhen People’s Hospital (The Second Clinical Medical College, Jinan University; The First Affiliated Hospital, Southern University of Science and Technology), Shenzhen Guangdong China; ^4^ Medical Laboratory Shenzhen Prevention and Treatment Center for Occupational Diseases Shenzhen China; ^5^ State Key Laboratory of Oncogenomics School of Chemical Biology and Biotechnology, Peking University Shenzhen Graduate School Shenzhen China; ^6^ Institute of New Drug Research, College of Pharmacy/Guangdong Province Key Laboratory of Pharmacodynamic Constituents of TCM and New Drugs Research/International Cooperative Laboratory of Traditional Chinese Medicine Modernization and Innovative Drug Development of Ministry of Education (MOE) of China Jinan University Guangzhou China; ^7^ State Key Laboratory of Biotherapy, West China Hospital, Sichuan University, and Collaborative Innovation Center for Biotherapy Sichuan University Chengdu China

**Keywords:** amyotrophic lateral sclerosis, derivative of arctigenin, inflammation, mitochondria, proteomics

## Abstract

**Aim:**

Amyotrophic lateral sclerosis (ALS) is a severe neurodegenerative disease characterized by progressive death of upper and lower motor neurons, leading to generalized muscle atrophy, paralysis, and even death. Mitochondrial damage and neuroinflammation play key roles in the pathogenesis of ALS. In the present study, the efficacy of A‐1, a derivative of arctigenin with AMP‐activated protein kinase (AMPK) and silent information regulator 1 (SIRT1) activation for ALS, was investigated.

**Methods:**

A‐1 at 33.3 mg/kg was administrated in SOD1^G93A^ transgenic mice orally from the 13th week for a 6‐week treatment period. Motor ability was assessed before terminal anesthesia. Muscle atrophy and fibrosis, motor neurons, astrocytes, and microglia in the spinal cord were evaluated by H&E, Masson, Sirius Red, Nissl, and immunohistochemistry staining. Protein expression was detected with proteomics analysis, Western blotting, and ELISA. Mitochondrial adenosine triphosphate (ATP) and malondialdehyde (MDA) levels were measured using an assay kit.

**Results:**

A‐1 administration in SOD1^G93A^ mice enhanced mobility, decreased skeletal muscle atrophy and fibrosis, mitigated loss of spinal motor neurons, and reduced glial activation. Additionally, A‐1 treatment improved mitochondrial function, evidenced by elevated ATP levels and increased expression of key mitochondrial‐related proteins. The A‐1 treatment group showed decreased levels of IL‐1β, pIκBα/IκBα, and pNF‐κB/NF‐κB.

**Conclusions:**

A‐1 treatment reduced motor neuron loss, improved gastrocnemius atrophy, and delayed ALS progression through the AMPK/SIRT1/PGC‐1α pathway, which promotes mitochondrial biogenesis. Furthermore, the AMPK/SIRT1/IL‐1β/NF‐κB pathway exerted neuroprotective effects by reducing neuroinflammation. These findings suggest A‐1 as a promising therapeutic approach for ALS.

## Graphical abstract

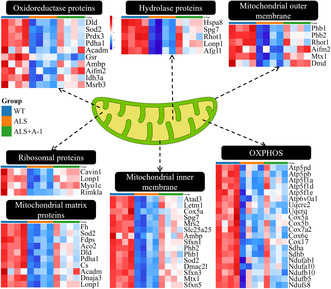

## 1 INTRODUCTION

Amyotrophic lateral sclerosis (ALS) is a progressive neurodegenerative disease first described in 1874 by Jean‐Martin Charcot, a Frenchman known as the father of neurology.^1^ It is characterized by progressive motor neuron death (both upper and lower motor neurons), resulting in muscle weakness, progressive paralysis, and death by respiratory failure within 3–5 years after onset.^2^ Among the types of ALS, sporadic (SALS) is more common, accounting for 90%–95% of cases, while the remaining 5%–10% are familial ALS (FALS).^3^

The etiology of ALS is currently unknown, but as with several other neurodegenerative diseases, mitochondrial dysfunction, and neuroinflammation have become increasingly compelling hypotheses in ALS research.^4^, ^5^, ^6^ AMP‐activated protein kinase (AMPK), a key molecule in the regulation of cellular energy metabolism, is mainly responsible for monitoring cellular inputs and outputs to maintain the normal operation of cellular physiological activities.^7^, ^8^ AMPK regulates silent information regulator 1 (SIRT1) and activated AMPK and SIRT1 upregulate the expression of peroxisome proliferator‐activated receptor‐γ coactivator‐1α (PGC‐1α) and promote mitochondrial biosynthesis via the AMPK/SIRT1/PGC‐1a signaling pathway.^9^, ^10^

**FIGURE 1:**
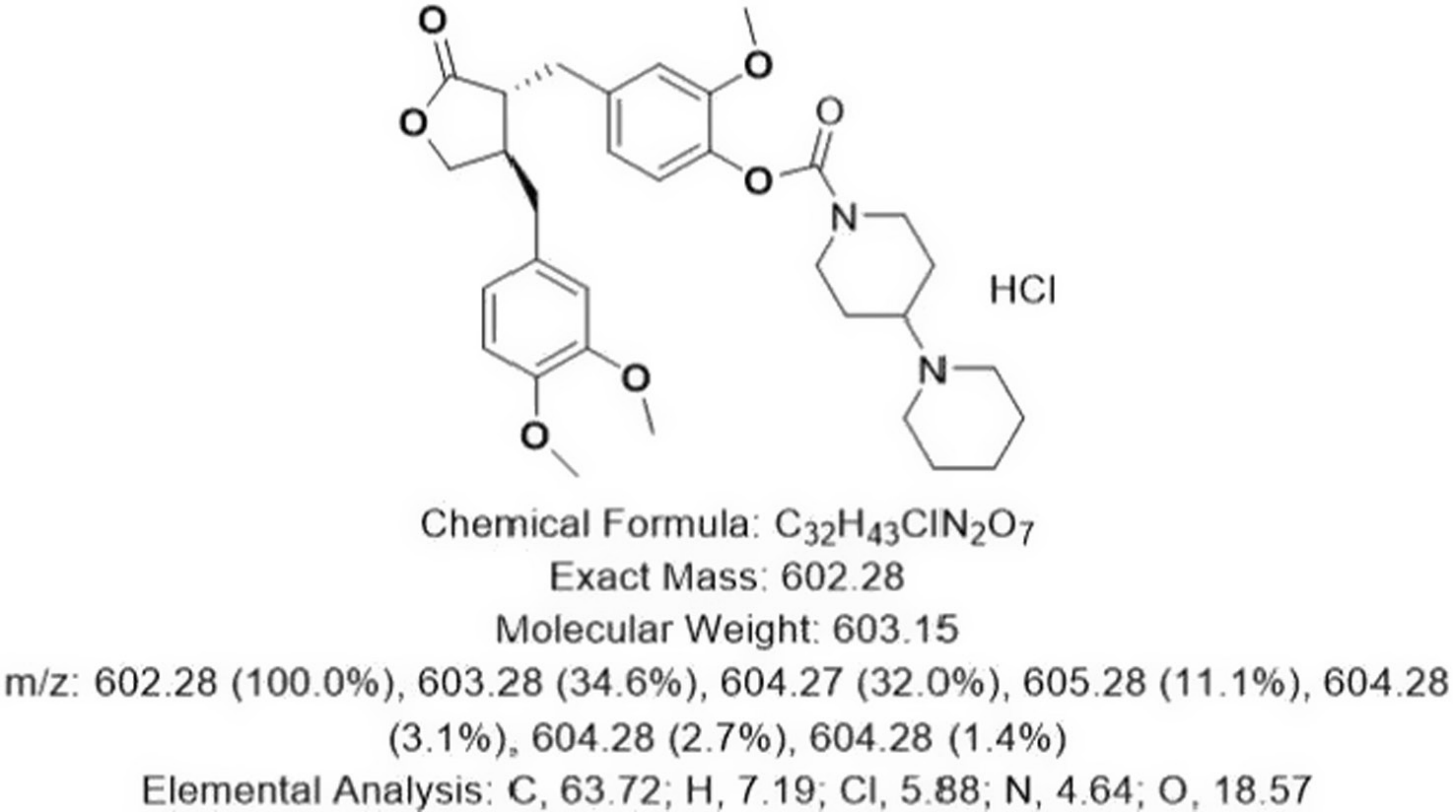
Chemical structure formula, chemical formula, exact mass, molecular weight, mass‐to‐charge ratio, and elemental analysis of A‐1.

Arctigenin (ATG) is a phenylpropanoid dibenzyl butyrolactone lignan. As an active ingredient of burdock, ATG has been proven to exert hypoglycemic, anti‐inflammatory, antioxidant, antibacterial, antiapoptotic, and antitumor functions.^11^, ^12^, ^13^ The A‐1 used in this study is a derivative of ATG, which increases the water solubility of ATG. Its chemical structure, formula, chemical formula, and other information are shown in Figure 1.

In the present study, we investigated the efficacy of oral administration of A‐1 in SOD1^G93A^ mice, a model of ALS, and further discussed the mechanism of the neuroprotective effect of A‐1 by improving mitochondrial function and inhibiting neuroinflammation.

## 2 METHODS

### 2.1 Animals and treatments

In this study, female B6SJL Tg‐SOD1*G93A‐1Gur/J mice carrying the human SOD1 mutant, obtained from Jackson Laboratories (Bar Harbor, ME, USA), were used. The mice were housed at the Experimental Animal Center at Peking University, Shenzhen Graduate School, in a room with a 12‐h light–dark cycle, constant temperature and humidity, and free access to water and food.

To investigate the effects of A‐1 treatment on mice carrying the human SOD1 mutant, the mice were randomly divided into three groups of eight mice each according to genotype. Nontransgenic mice from the same litter were used as the control group, and mice carrying the human SOD1 mutant were divided into model and treatment groups. All mice were 13 weeks old at the start of the experiment.

During the 6‐week treatment period, the treatment group was administered A‐1 at a dosage of 33.3 mg/kg, while the model and control groups were given the same volume of saline once a day at the same time. After 6 weeks of treatment, the mice were behaviorally tested, followed by terminal anesthesia.

During the animal experimentation, we followed the ARRIVE guidelines and conducted the study in accordance with the UK Animals (Scientific Procedures) Act 1986 and relevant guidance, as well as the National Research Council Guide for the Care and Use of Laboratory Animals. Moreover, The Animal Care and Use Committee of the Experimental Animal Center at Peking University, Shenzhen Graduate School, approved the animal experiments. The approval number for the animal experiment is ER‐0013‐005.

### 2.2 Behavioral tests

#### 2.2.1 Grip strength test

Paw grip strength was measured to evaluate the muscular strength in the limbs of mice. The mice were carefully positioned in the middle of a gripping plate, and once they had securely grasped it, they were gently pulled by the tail with consistent force. This test was conducted three times per animal, and the highest result was recorded as the final assessment value.

#### 2.2.2 Hanging endurance test

A metal net was employed to evaluate the grasping force of the limbs of the mice. Each animal was placed at the center of a metal net measuring 60 cm × 40 cm. Once the mice had grasped the metal mesh, it was rotated by 180 degrees to an inverted level, and the time that the mice hung on the metal grid was recorded.

Prior to testing, all mice underwent training, with a predetermined maximum test time of 180 s. Each animal was subjected to two repeated experiments, and the average endurance time of each mouse was obtained as the evaluation value.

#### 2.2.3 Climbing‐pole test

The pole climbing test was used to assess the motor and coordination abilities of the mice. The experiment used a homemade wooden pole approximately 50 cm long and 1 cm in diameter wrapped with medical gauze to increase friction. The mice were placed head down on top of the vertical pole, and the time of descent from the top of the pole to the bottom platform was recorded. Before testing, each mouse was trained for 3 consecutive days twice a day, with a maximum cutoff value of 15 s. The experimental procedure was repeated three times, and the average crawling time of each mouse was calculated as the evaluation value.

#### 2.2.4 Rotarod fatigue test

The rotarod fatigue test was used to assess endurance, balance, and coordination in mice.^14^ Each mouse was carefully positioned on the rotating bar of a rotating bar fatigue tester, with a maximum rotation speed of 25 revolutions per minute. Prior to the commencement of the formal experiment, each mouse underwent a rigorous training regimen, which spanned 3 days and was conducted twice a day, with a cutoff value of 180 s. The formal experiment was replicated thrice, with a minimum intertest interval of 1 h. The average duration of adherence exhibited by each mouse was subsequently calculated and taken as the measure of evaluation.

#### 2.2.5 Gait test

The mouse gait test experiment was conducted by using a walking tracking analysis system (Framework 4.0) for mouse gait acquisition. The experimental mice were placed in a wiped clean light‐proof walking channel (width approximately 5 cm), the video acquisition system was underneath the walking channel and aimed at the channel through which the mice passed, and the walking channel could show the mice's footprints. The mice were allowed to enter the walking channel naturally during the experiment. The high‐speed camera recorded at least three consecutive walking cycles of video for subsequent data analysis, and the mice could not have a stopping time in the middle; otherwise, the mice's walking video was rerecorded.

### 2.3 Proteomics

#### 2.3.1 Protein extraction and enzymatic digestion

The L4‐L5 spinal cord segments of each group of animals were collected, with four samples per group, immediately placed in a −80°C ultralow temperature freezer and stored until use. During the experiment, the tissue was placed in a mixed solution containing 8 M urea, PBS (pH 8.0), and protease and phosphatase inhibitors and sonicated at low temperature (Sonics VCX‐150, Newton, Connecticut, USA). After the samples were sonicated, they were centrifuged at 12,000 rpm for 30 min at 4°C to allow cell debris and other materials to settle. The supernatant was carefully aspirated and transferred to a new EP tube. The protein concentration of the supernatant was determined by the BCA protein quantification assay kit (Thermo Fisher, New Jersey, USA) after appropriate dilution and 50 μg of protein was used for subsequent experiments in each sample. Dithiothreitol (DTT) was added to each sample at a final concentration of 10 mM, mixed well, and incubated for 1 h in a dry heat incubator at 55°C, followed by the addition of 25 mM iodoacetamide (IAA) and incubation for 1 h at room temperature in the dark. Then, trypsin (1:25 w/w) (Promega, WI, USA) was added to the samples, which were digested for 4 h at 37°C, and the urea concentration in the samples was diluted to 1.0 M with PBS (pH 8.0) and further digested for 11 h in a constant temperature incubator. After digestion, the pH of the sample solution was adjusted to 1–2 with trifluoroacetic acid (TFA), and the sample was desalted using an OASIS HLB column (Waters, USA). Finally, the sample was dried in a vacuum centrifuge at low temperature and labeled with tandem mass tags (TMTs).

#### 2.3.2 TMT labeling and LC–MS/MS analysis

A total of 50 μL of 200 mM triethylammonium bicarbonate (TEAB) was added to the dried sample and resuspend. Then, the peptides were labeled with TMT reagents at room temperature for 1 h, followed by quenching with 5% hydroxylamine for 15 min. The differentially labeled samples were mixed, desalted using a desalting column, vacuum centrifuged dry at low temperature, and then resuspended in 100 μL of 0.1% formic acid. The labeled peptides were loaded onto an Xbridge BEH300 C18 column (Waters, USA), separated using Extreme 3000 UHPLC (Thermo Fisher Scientific, USA), and divided into 60 fractions according to the manual. All fractions were vacuum centrifuge dried at low temperature, resuspended in 20 μL of 0.1% formic acid, and analyzed by liquid chromatography (LC)–mass spectrometry (MS)/MS. Finally, data were retrieved from the UniProt mouse Fasta database using protein discovery software. The relative protein expression levels were calculated based on the reported ion intensities of each peptide. The differential protein expression in the control group WT, SOD1^G93A^ model, and A‐1 treated SOD1^G93A^ mice was set at *p* < 0.05 for any two groups using the *t*‐test method in Perseus software.

#### 2.3.3 Bioinformatics analysis

We first used Perseus software to screen for differentially expressed proteins in the SOD1^G93A^ model and A‐1‐treated SOD1^G93A^ mice. The abundance of each differentially expressed protein was evaluated using heatmap analysis. Protein clustering analysis was performed using DAVID Bioinformatics Resource 6.8 (https://david.ncifcrf.gov/) to determine the biological processes and pathways of differentially expressed proteins in the A‐1‐ and vehicle‐treated animal groups, which was also used for gene ontology analysis. The molecular complex detection (MCODE) algorithm was utilized to identify densely connected protein–protein interaction (PPI) regions. Image visualization processing was performed using the Hiplot website (https://hiplot.com.cn/) and Cytoscape software.

### 2.4 Western blotting

The spinal cord tissue was subjected to ultrasonic lysis using 8 M urea lysis buffer containing 1× protease and phosphatase inhibitors (Thermo Scientific, NJ, USA). The extracted protein concentration was determined using the BCA protein quantification assay kit (Thermo Scientific, USA). Equal amounts of protein samples were mixed with loading buffer and heated at 99°C in a metal bath for 10 min. The proteins were separated on 8%–12% SDS–PAGE gels and transferred onto 0.45 μm polyvinylidene fluoride (PVDF) membranes. The membranes were then blocked with 5% skim milk at room temperature for 1.5 h. Subsequently, the blocked membranes were incubated overnight at 4°C with appropriately diluted primary antibodies (the antibody information used is shown in Table S3). After washing off the unbound primary antibodies from the PVDF membranes using TBST buffer (3 times, 10 min each time), the membranes were incubated with the corresponding secondary antibodies at room temperature for 1.5 h on a shaker. Finally, the target protein grayscale values were analyzed using ImageJ software following exposure with an ECL kit (Thermo Scientific, NJ, USA).

### 2.5 Staining for muscle pathology

Following anesthesia, the mice were perfused with saline through the heart. The gastrocnemius muscle from one hind limb was carefully excised and subsequently fixed in 4% paraformaldehyde (pH 7.4) for 2 days, followed by dehydration and embedding. Serial sections of tissue (5 μm) were stained with hematoxylin–eosin (H&E) or Masson staining (8 mice per group). Digital images of the stained sections were captured using a scanning microscope imaging system. The level of muscle tissue fibrosis was determined by calculating the percentage of blue areas over the entire tissue area using ImageJ analysis software, which was obtained through Masson staining.

### 2.6 Nissl staining

The mice were anesthetized by intraperitoneal injection of 1% pentobarbital sodium, and the thorax was opened. The heart was perfused with physiological saline solution. At the end of the experiment, the mice were anesthetized with 1% sodium pentobarbital intraperitoneally; then, the thoracic cavity was opened, and the heart was perfused with saline. The fourth to fifth segments of the lumbar spine were removed, fixed in 4% paraformaldehyde for 48 h, dehydrated, and paraffin‐embedded. Sections (thickness 5 μm) were routinely dewaxed and rehydrated, and then the sections were placed in Nissl staining solution, stained for 10 min at room temperature, dehydrated, and sealed with neutral resin. The stained images were captured by a digital pathology scanning system. Motor neurons were counted on the side of the anterior horn of the spinal cord. Motor neurons were counted on one side of the ventral horn of the spinal cord. Only those with a diameter greater than 20 microns and with clearly discernible cell nuclei, nucleoli, and cytoplasm were included in the statistical analysis.

### 2.7 Immunohistochemistry

Spinal cord slices were routinely deparaffinized and rehydrated, followed by antigen retrieval using sodium citrate buffer by boiling for 10 min. After natural cooling, the slices were washed for 15 min on a shaker with PBS and then treated with hydrogen peroxide block for 15 min to remove endogenous peroxide. The slices were then incubated at room temperature with a protein block for 1 h. Subsequently, they were incubated overnight at 4°C with primary antibodies against GFAP and Iba1 (diluted 1:200). After incubation with the primary antibodies, the slices were incubated for 1 h at room temperature with biotinylated goat anti‐polyvalent, followed by incubation with streptavidin peroxidase for 30 min. Finally, the slices were stained with DAB, dehydrated with an ethanol gradient, and cleared with xylene. Images were captured using a digital pathology scanning system.

### 2.8 Enzyme‐linked immunosorbent assay (ELISA)

Proteins were extracted from the lumbar spinal cord of each mouse group using 8 M urea lysis buffer containing protease and phosphatase inhibitors. The supernatant was collected after centrifugation at 12,000 rpm for 30 min at 4°C using a low‐temperature centrifuge. The expression levels of IL‐6 and IL‐1β in the samples were measured using a mouse ELISA kit (Elabscience, Wuhan, China) following the manufacturer's instructions. The absorbance was read at 450 μm using an enzyme marker (BioTek, Winooski, VT, USA), and the expression levels were calculated according to the standard curve. Inflammatory factor levels were calculated in pg/μg protein.

### 2.9 Mitochondrial adenosine triphosphate levels

Adenosine triphosphate levels in spinal cord tissues were measured using an adenosine triphosphate assay kit (Beyotime, Haimen, China). Briefly, proteins were extracted with ATP lysate, a portion was used for BCA quantification, a portion was centrifuged at high speed (12,000 rpm) for 30 min after a metal bath at 100°C, and the supernatant (100 μL) was mixed with ATP assay solution (100 μL) in a 96‐well black plate and placed at room temperature for 3 min. ATP levels were read using a microplate reader with luminometer function. The ATP level was quantified in nmol/mg protein.

### 2.10 Malondialdehyde (MDA) content determination

The malondialdehyde (MDA) content in the spinal cord tissues of each group of mice was determined using an MDA detection kit (Nanjing Jiancheng Bioengineering Institute, China). Briefly, the protein concentration of the samples was measured first, and then the corresponding reagents were added. The samples were heated in a water bath at 95°C for 40 min, followed by cooling with running water and centrifugation at 4000 rpm for 10 min. Finally, 200 μL of the supernatant was taken and added to a 96‐well plate, and the absorbance was measured at 532 nm using an enzyme‐linked immunosorbent assay reader. The MDA level was expressed as nmol/mg protein.

### 2.11 Statistical analysis

Data are expressed as mean ± SEM and analyzed with GraphPad Prism 8.0 (GraphPad Software, Inc., La Jolla, CA, USA). All data were tested by Shapiro–Wilk or Kolmogorov–Smirnov normality test. Data followed a normal distribution and were analyzed using two‐tailed Student's *t*‐test, one‐way ANOVA with Tukey's post hoc test. Otherwise, data were analyzed using the Mann–Whitney test. *p* values less than 0.05 were considered statistically significant.

## 3 RESULTS

### 3.1 A‐1 administration improved motor performance in SOD1^G93A^ mice

**FIGURE 2:**
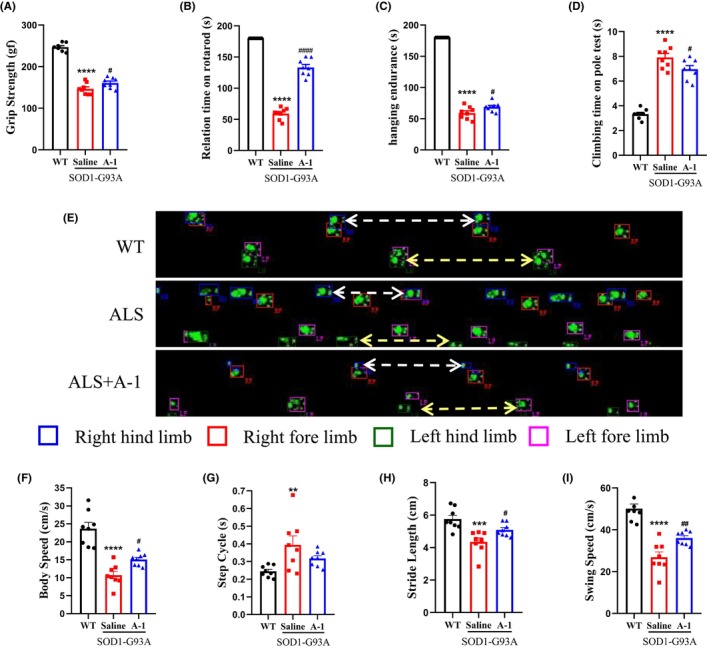
Administration of A‐1 improved motor performance in SOD1^G93A^ mice. Thirteen‐week‐old SOD1^G93A^ mice were administered A‐1 for 6 weeks, and then, motor performance was detected. (A) Grip strength measured by the grip strength test, (B) retention time on the rotarod by the rotarod test, (C) hanging endurance time detected with the hanging endurance test, (D) climbing time on pole tests by climbing‐pole test, and (E) representative footprints of each group of mice are shown. The footprints of the four paws are indicated by different colored boxes, as shown in the legend on the lower side of the footprints. (F–I) Body movement speed, step frequency of hind limbs, stride length, and swing speed are shown. Data are shown as the mean ± SEM. **p < 0.01, ***p < 0.001, ****p < 0.0001 versus WT group. ^#^ *p* < 0.05, ^##^ *p* < 0.01, ^####^ *p* < 0.0001 versus SOD1^G93A^ saline group. *n* = 8 for each group.

Pole climbing, stick turning, suspension, grip, and gait were used to evaluate the locomotor ability of mice in terms of balance control, endurance, coordination, and muscle strength. Compared with the WT mice, the SOD1^G93A^ mice showed a significant decrease in grip strength, time spent on the rotarod, hanging endurance, and climbing time in the pole test, and A‐1 administration significantly improved motor performance in SOD1^G93A^ mice (Figure 2A–D). The gait test results showed that SOD1^G93A^ mice had disorganized walking footprints, uncoordinated limbs, and unsteady walking compared with WT mice, and A‐1 administration improved gait disorders in SOD1^G93A^ mice (Figure 2E). Step frequency, stride length, and walking speed were significantly decreased, and the walking cycle was significantly prolonged in SOD1^G93A^ mice compared with WT mice. A‐1 treatment remarkably reversed these motor dysfunctions (Figure 2F–I).

### 3.2 A‐1 administration attenuated pathological manifestations in SOD1^G93A^ mice

**FIGURE 3:**
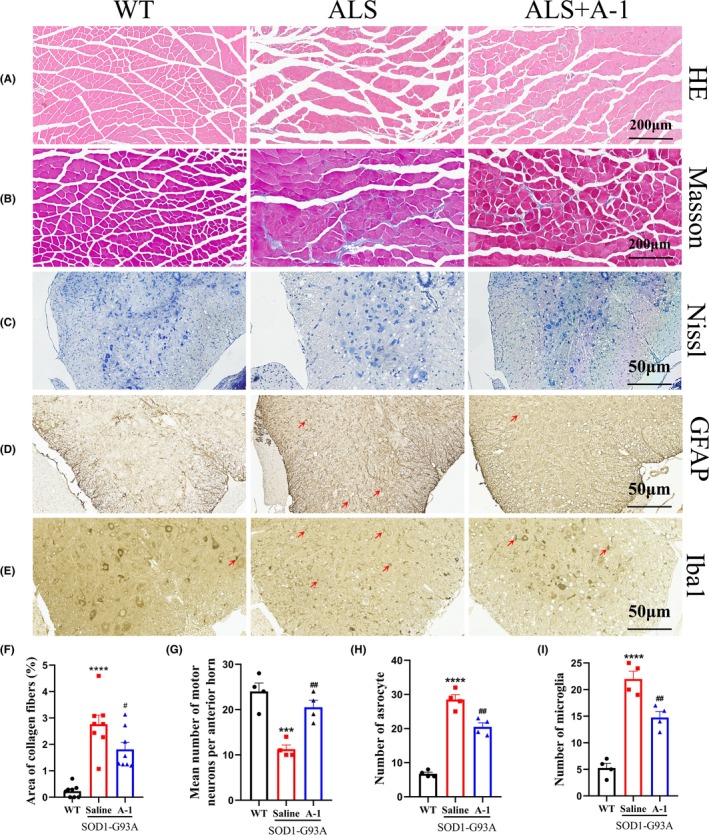
A‐1 treatment attenuated the pathological manifestations in SOD1^G93A^ mice. (A) Representative images of H&E staining. (B) Representative images of Masson staining with collagen fibers in blue. (C) Representative images of Nissl staining of the L4‐L5 segment of the spinal cord. Representative images of immunohistochemical staining of (D) astrocytes (GFAP) and (E) microglia (Iba1) in the spinal cord. (F) Quantification of the area of collagen fibers by Masson staining. (G) Quantitative analysis of motor neurons in the anterior horn of the spinal cord. (H, I) Quantification of astrocytes and microglia in the spinal cord. Data are shown as the mean ± SEM. ***p < 0.001, ****p < 0.0001 versus WT group. ^#^ *p* < 0.05, ^##^ *p* < 0.01 versus SOD1^G93A^ saline group. Gastrocnemius pathology staining *n* = 8 for each group, and spinal cord pathology staining *n* = 4 for each group. Red arrows indicate activated astrocytes or microglia.

Compared with WT mice, SOD1^G93A^ mice displayed significant atrophy and fibrosis of the gastrocnemius muscle and reduced motor neurons in the spinal cord (Figure 3), while A‐1 significantly slowed the atrophy of the gastrocnemius muscle in ALS mice (Figure 3A), reduced the area of fibrosis in the gastrocnemius muscle (Figure 3B,F), and decreased the loss of motor neurons in SOD1^G93A^ mice (Figure 3C,G).

The numbers of astrocytes and microglia in the L4‐L5 segment of the spinal cord were detected using immunohistochemistry. Compared with that in WT mice, the number of activated astrocytes and microglia in the spinal cord of SOD1^G93A^ mice was significantly increased, and A‐1 administration reduced the activation of astrocytes (Figure 3D,H) and microglia (Figure 3E,I) in the spinal cord of SOD1^G93A^ mice.

### 3.3 A‐1 administration altered the protein expression profile of SOD1^G93A^ mice

**FIGURE 4:**
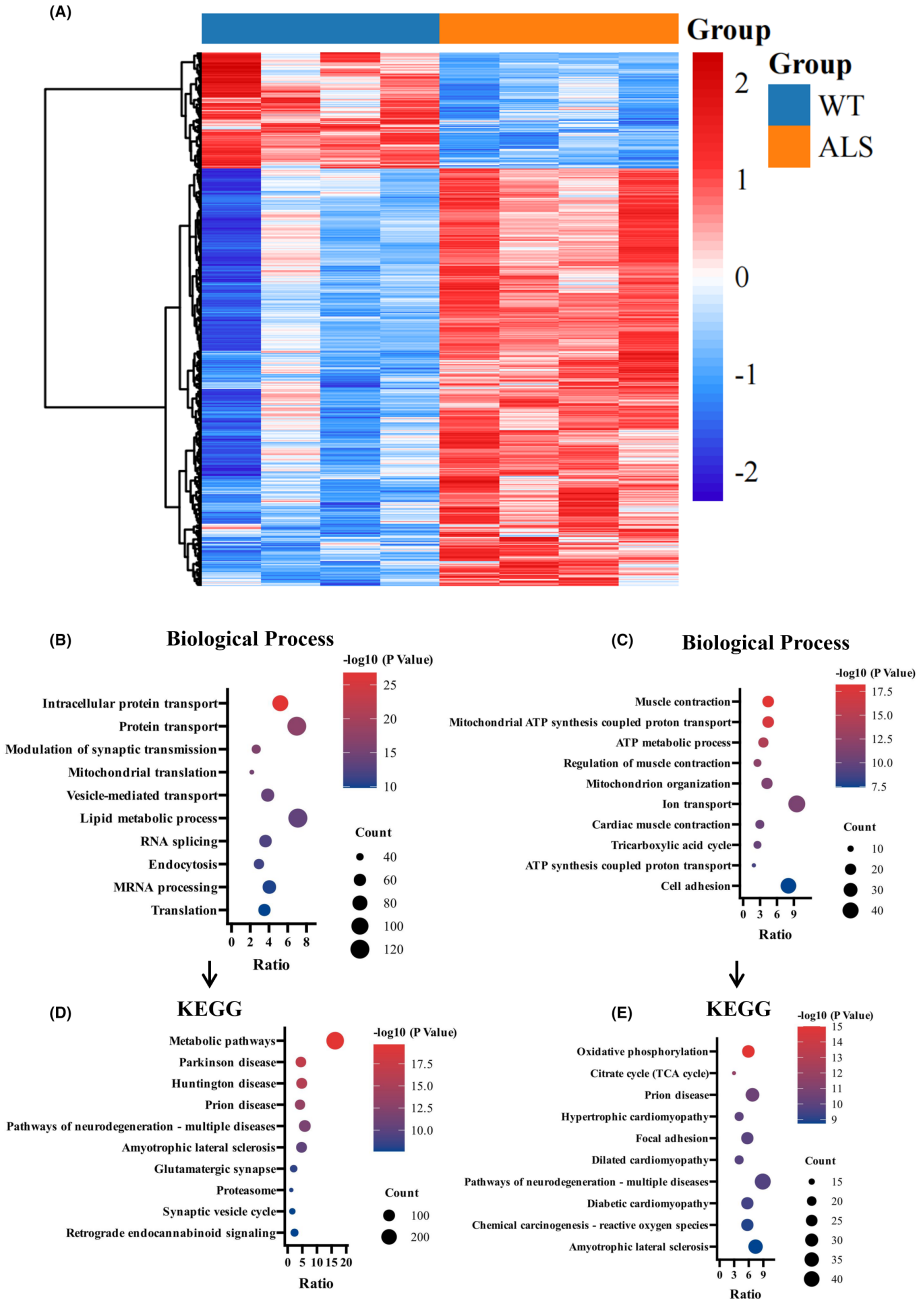
Proteomic analysis of differentially expressed (DE) proteins in the spinal cords of WT mice and ALS mice. (A) Heatmap expression profiles of DE proteins in the spinal cords of model and control mice. (B) Biological processes involving the upregulated DE proteins. (C) Biological processes involving the downregulated DE proteins. (D) KEGG pathways involved in the upregulated DE proteins. (E) KEGG pathways involved in the downregulated DE proteins.

Nontargeted proteomics and TMT markers in SOD1^G93A^ mice and WT mice were analyzed to characterize the abnormal protein networks. A total of 5376 proteins were detected in spinal cord tissue (false discovery rate [FDR] <1%). There were 2181 differentially expressed proteins in the spinal cord of SOD1^G93A^ mice compared to WT mice (Figure 4A). Bioinformatics analysis (https://david.ncifcrf.gov/) revealed that the upregulated proteins were enriched in biological processes such as intracellular protein transport, mitochondrial translation, vesicle‐mediated transport, and regulation of synaptic transmission (Figure 4B), and the corresponding pathways were enriched in metabolic pathways, Parkinson's disease, and neurodegenerative pathways (Figure 4C). The downregulated proteins were mainly enriched in muscle contraction, ATP synthesis, and mitochondrial organization (Figure 4D), and the corresponding pathways were enriched in oxidative phosphorylation, tricarboxylic acid cycle (TCA cycle), and focal adhesion pathways (Figure 4E). ALS mice exhibited dysfunction in synaptic function, muscle contraction, and aerobic respiration.

**FIGURE 5:**
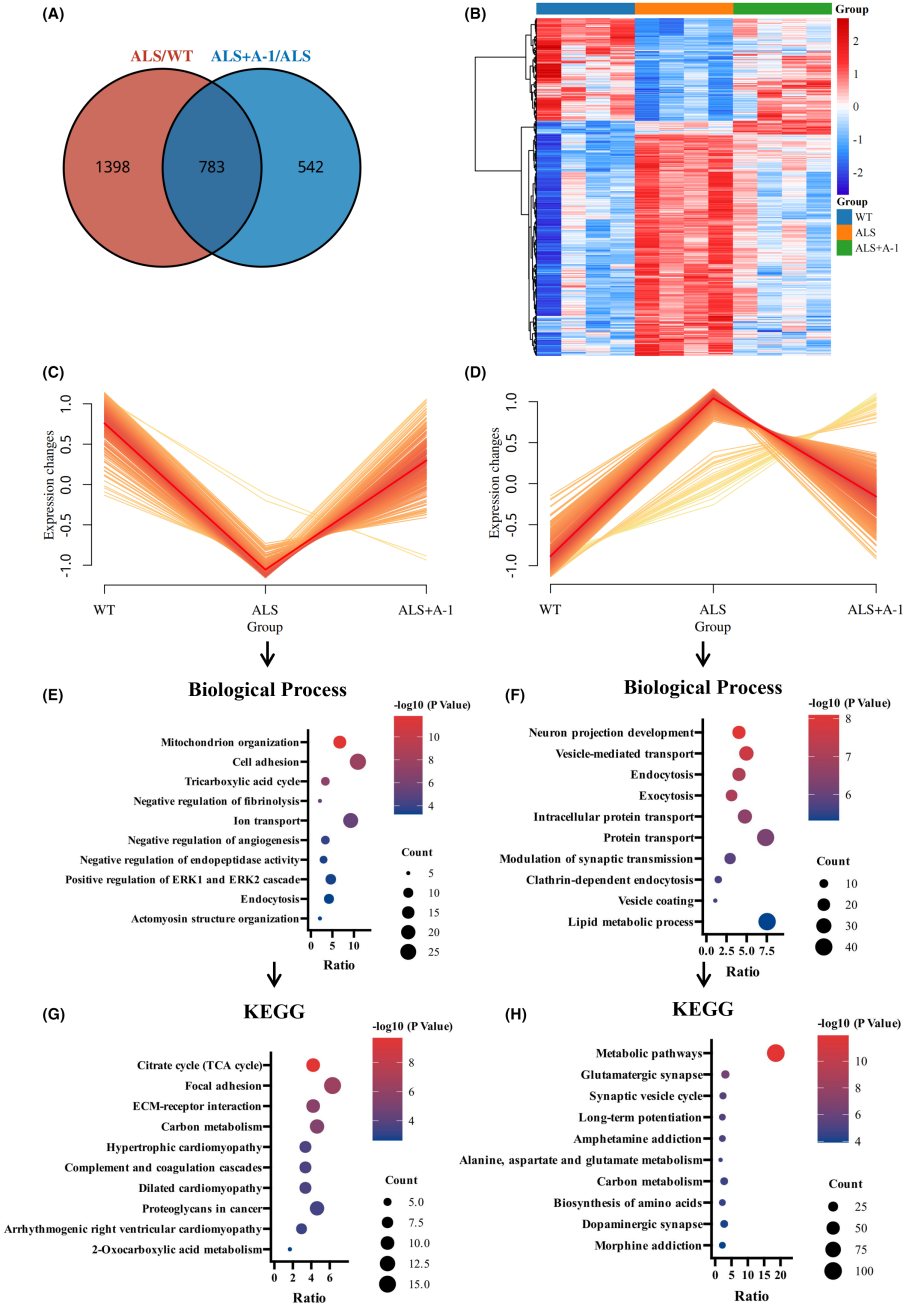
Differential protein analysis of spinal cord proteomics in three groups of mice. (A) Venn diagram of DE proteins in the SOD1^G93A^ model and A‐1 treatment. (B) Heatmap expression profiles of spinal cord DE proteins. Two improved expression patterns of differential proteins in proteomics (C, D) and corresponding top 10 biological processes (E, F) and KEGG pathways (G, H).

A total of 1325 proteins were differentially expressed in the spinal cord of SOD1^G93A^ mice with or without A‐1 administration, 783 of which overlapped with the differential proteins between WT mice and SOD1^G93A^ mice (Figure 5A,B). A total of 783 differentially expressed proteins were classified into two categories by clustering analysis (Figure 5C,D). The upregulated proteins were mainly involved in biological processes such as mitochondrial organization, cell adhesion, and the tricarboxylic acid cycle (Figure 5E), and the pathways involving the related proteins were mainly the citrate cycle (TCA cycle), focal adhesion, and ECM‐receptor interaction (Figure 5G). While the downregulated proteins were mainly involved in neuron projection development, vesicle‐mediated transport, endocytosis, and other biological processes (Figure 5F), the pathways involved in the related proteins were mainly metabolic pathways, glutamatergic synapses, and synaptic vesicle cycles (Figure 5H).

**FIGURE 6:**
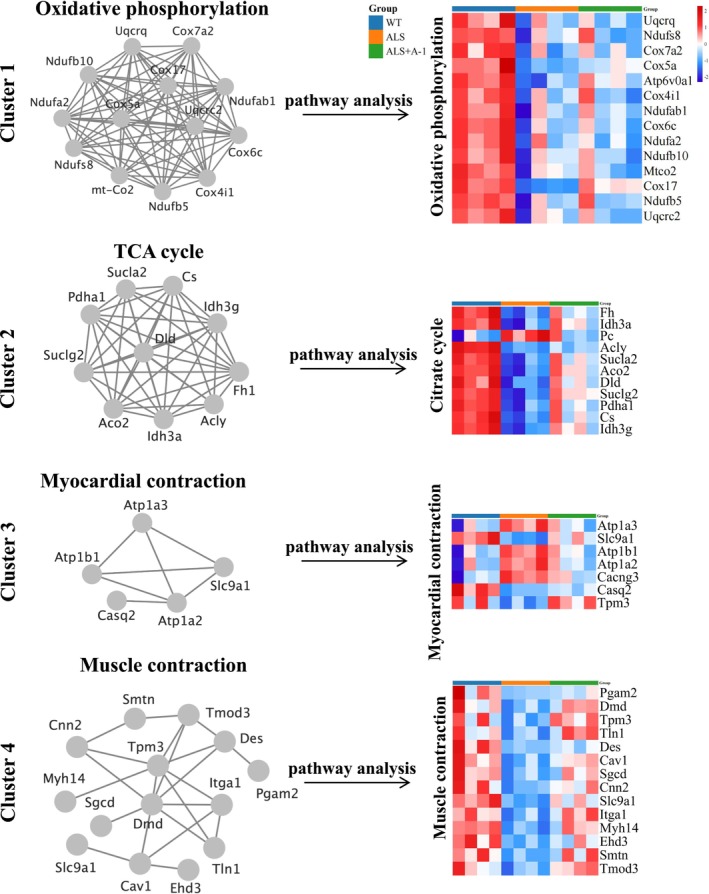
Molecular complex assay (MCODE) analysis to find core modules in the protein–protein interaction (PPI) network, followed by functional annotation and pathway analysis in four major improved clusters by WebGestalt.

A molecular complex assay (MCODE) was performed to identify the core set of differentially expressed protein genes after A‐1 administration. The core set genes of Cluster 1 were related to oxidative phosphorylation, with the oxidative phosphorylation pathway being the implicated pathway. The core set genes of Cluster 2 were related to the tricarboxylic acid cycle. The core gene set of Cluster 3 was associated with myocardial contraction and involved in the myocardial contraction pathway. The core gene set of Cluster 4 was associated with muscle contraction and involved in the muscle contraction pathway (Figure 6).

### 3.4 A‐1 administration ameliorated mitochondrial dysfunction in SOD1^G93A^ mice

A‐1 upregulated oxidative phosphorylated proteins, including Ndufs8 (NADH dehydrogenase [ubiquinone] iron–sulfur protein 8, mitochondrial), Ndufb5 (NADH dehydrogenase [ubiquinone] 1 beta subcomplex subunit 5, mitochondrial), Ndufab1 (acyl carrier protein, mitochondrial), and cox5a (cytochrome c oxidase subunit 5A, mitochondrial) (Figure 6), which implied that A‐1 might play a role in improving mitochondrial function.

**FIGURE 7:**
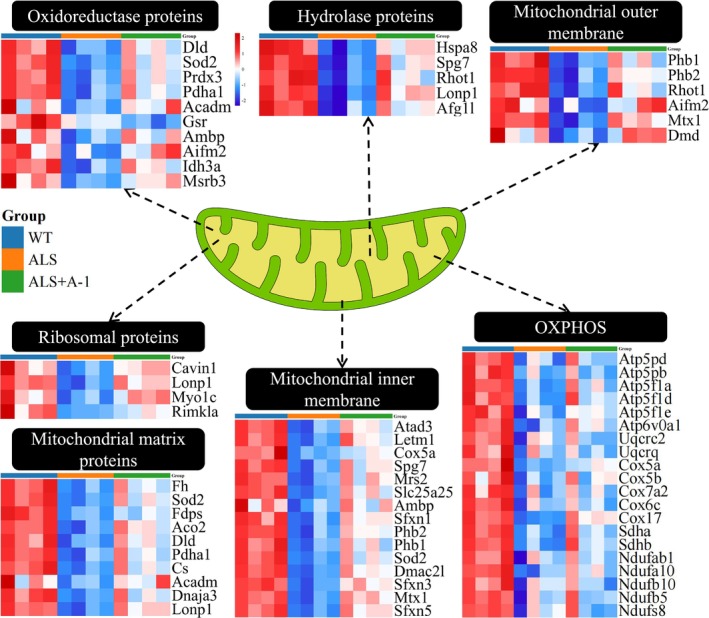
The expression profiles of differentially expressed mitochondrial proteins in the spinal cord. The Hiplot website (https://hiplot.com.cn/) was used to normalize the expression values of each protein in the three groups of mice. Then, these data were used to plot mitochondrial heatmaps. Expression differences were set at *p* < 0.05. Red indicates upregulation, and blue indicates downregulation.

Following administration of A‐1 in SOD1^G93A^ mice, an upregulation trend in the majority of differentially expressed mitochondrial proteins was observed. The involved proteins included outer mitochondrial membrane, inner mitochondrial membrane, mitochondrial matrix, oxidative phosphorylation, hydrolase proteins, ribosomal proteins, and oxidoreductase proteins, of which those related to mitochondria included ATP synthase peripheral stalk subunit d, cytochrome c oxidase subunit 5A, and NADH dehydrogenase [ubiquinone] 1 alpha subcomplex subunit 10 (Figure 7). This finding implied that A‐1 had a protective effect on mitochondria during ALS progression.

**FIGURE 8:**
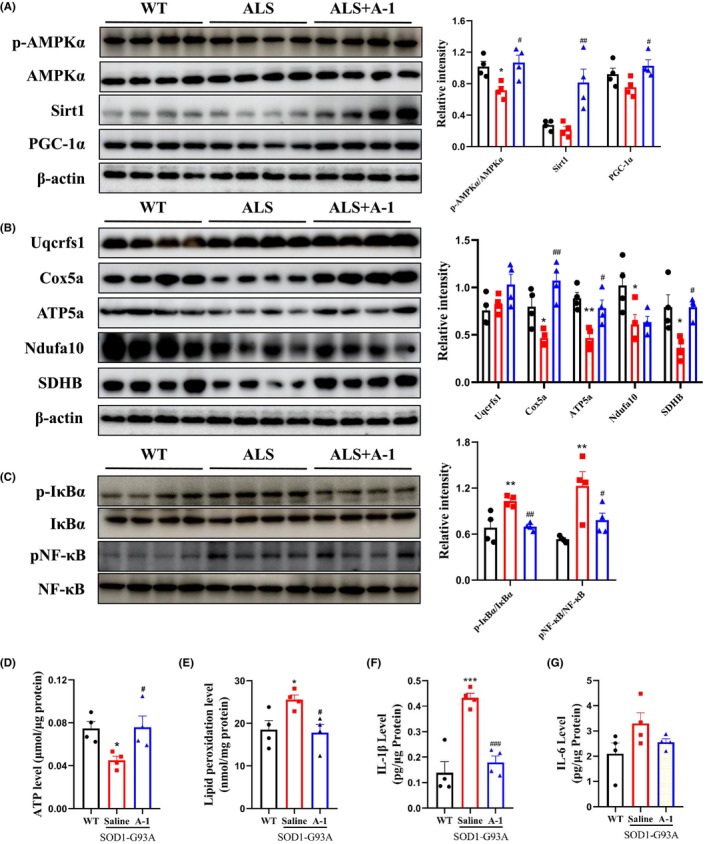
A‐1 activated AMPK, improved mitochondrial function, and suppressed inflammatory responses. (A) Expression levels and quantification of p‐AMPKα, AMPKα, Sirt1, and PGC‐1α. (B) Expression levels of the electron transport chain proteins Uqcrfs1, Cox5a, ATP5a, Ndufa10, and SDHB were detected by Western blotting and quantitative analysis. (C) Expression levels and quantification of p‐IκBα, IκBα, NF‐κB, and pNF‐κB. (D) ATP levels in the spinal cord were measured by an ATP assay kit. (E) Measurement of lipid peroxidation levels in the spinal cord with a malondialdehyde (MDA) assay kit. (F, G) ELISAs for IL‐1β levels and IL‐6 levels in the spinal cord. Data are shown as the mean ± SEM. *p < 0.05, **p < 0.01, ***p < 0.001 versus WT group. ^#^ *p* < 0.05, ^##^ *p* < 0.001, ^###^ *p* < 0.001 versus SOD1^G93A^ saline group. *n* = 4 for each group.

A‐1 administration activated spinal AMPK and increased the expression of proteins in downstream pathways, such as silent mating type information regulation 2 homolog‐1 (SIRT1) and the protein peroxisome proliferator‐activated receptor γ coactivator 1‐α (PGC‐1α), in the mitochondrial biogenesis pathway (Figure 8A) (*p* < 0.01, *p* < 0.05). Consistent with the data on mitochondrial biogenesis, the levels of mitochondrial electron transport chain proteins such as succinate dehydrogenase (SDHB), cytochrome C oxidase subunit 5a (Cox5a), adenosine triphosphate synthase subunit α (ATP5a) and adenosine triphosphate were significantly increased (Figure 8B,D). Compared with those in the WT mouse group, IκBα, and NF‐κB were activated, and the levels of the proinflammatory factors IL‐1β and IL‐6 were significantly increased in the spinal cord tissue of SOD1^G93A^ mice, while A‐1 treatment decreased the levels of p‐IκBα, pNF‐κB, IL‐1β, and IL‐6 in the spinal cord tissue of SOD1^G93A^ mice (Figure 8C,E–G). In summary, A‐1 attenuated mitochondrial dysfunction and suppressed neuroinflammation in SOD1^G93A^ mice.

## 4 DISCUSSION

ALS is a serious neurodegenerative disease. To date, only riluzole and edaravone have been used clinically to treat ALS patients. However, the therapeutic efficiency is quite limited.^15^, ^16^ In the present study, we found that the small molecule Compound A‐1 slowed the progression of motor system dysfunction and the process of gastrocnemius atrophy, attenuated the degree of fibrosis in the gastrocnemius muscle, reduced the loss of spinal motor neurons, decreased the activation of microglia and astrocytes, and modified the abnormal protein profile, especially the AMPK/SIRT1/PGC‐1α pathway and AMPK/SIRT1/IL‐1β/NF‐κB pathway, in the spinal cord of SOD1^G93A^ mice. Our beneficial effects of A‐1 treatment provide a novel therapeutic strategy for ALS treatment.

Reduced mitochondrial function and altered mitochondrial morphology were reported in the postmortem tissues of ALS patients and ALS animal models.^17^ Therefore, mitochondria have received increasing attention and are considered potential therapeutic targets for ALS. Mitochondria control not only cellular energy production but also calcium homeostasis and apoptosis.^18^

An increasing number of studies have demonstrated that ATG has an important role in protecting the central nervous system and improving mitochondrial function via activation of AMPK and SIRT1. ATG promotes mitochondrial biogenesis and fatty acid synthesis and oxidation by activating AMPK, leading to enhanced endurance in mice.^19^ Overexpression of SIRT1 has been found to reduce the inflammatory response via nuclear factor‐κB (NF‐κB) and promote mitochondrial biosynthesis via the AMPK/SIRT1/PGC‐1a signaling pathway.^20^, ^21^ ATG exhibits neuroprotective effects against cerebral ischemia by inhibiting NLRP3 via SIRT1.^22^ However, there have been no reports about the therapeutic effect and mechanism of ATG or A‐1 on ALS.

A‐1 is a derivative of ATG with high water solubility. It is noteworthy that A‐1 can be metabolized into ATG after oral absorption. Our previous pharmacokinetic study conducted in SD rats revealed that the blood concentration of ATG within the 0–1 h interval after oral administration of A‐1 exceeds that of ATG administration with equimolar dosage (detailed pharmacokinetic data can be found in Tables S1 and S2, which are provided in the Supplementary Materials).

In the present study, A‐1 improved mitochondrial function manifested as a reduction in spinal ATP decline, restoration of electron transport chain‐related protein expression, and an increase in mitochondrial complex II (SDHB) and complex V (ATP5a) in the spinal cord in SOD1^G93A^ mice. A‐1 also activated AMPK and SIRT1. AMPK is the key molecule in the regulation of cellular energy metabolism,^23^ and it regulates SIRT1, which is involved in various physiological processes, such as cellular senescence, gene transcription, energy homeostasis, and the regulation of oxidative stress.^24^, ^25^ It has been found that activated AMPK and SIRT1 upregulate the expression of peroxisome proliferator‐activated receptor‐γ coactivator‐1α (PGC‐1α).^26^ PGC‐1α is a major regulator of mitochondrial biogenesis, which has been proven to improve motor neuron function and survival in ALS mice.^27^ In the present study, A‐1 treatment significantly increased the expression level of PGC‐1α in the spinal cord of SOD1^G93A^ mice. These results suggested that A‐1 treatment improved mitochondrial function by activating AMPK/SIRT1/PGC‐1α, which in turn increased mitochondrial biogenesis and further delayed the progression of ALS disease.

Astrocytes have toxic effects on motor neurons in patients with familial and sporadic ALS,^28^ and glial cell proliferation and microglial activation are among the main features of neuroinflammation in ALS.^29^, ^30^ Consistent with previous findings,^31^, ^32^ we found that astrocytic stromal cells and microglia were activated in the spinal cord of ALS mice. In general, astrocytes and microglia are neuroprotective; however, during disease progression, microglia shift from the M2 phenotype to the M1 phenotype, resulting in the production and release of proinflammatory cytokines and reactive oxygen species (ROS), which are toxic to neurons and ultimately lead to motor neuron damage and death.^33^ Activation of astrocytes increases the release of neurotoxic factors and decreases the release of neurotrophic factors, leading to neurological damage.^34^ Previous studies have demonstrated that ATG attenuates inflammation in colitis by upregulating SIRT1 and suppressing NLRP3 and downstream IL‐1β expression,^35^ alleviates acute lung injury (ALI) induced by lipopolysaccharide (LPS) by activating AMPK and inhibiting the NF‐κB signaling pathway,^36^ and improves memory impairment in a mouse model of Alzheimer's disease by decreasing the pathological accumulation of aggregated amyloid‐beta (Aβ) via inhibition of the AKT/mTOR axis while activating AMPK.^37^ As a derivative of ATG, A‐1 treatment significantly inhibited the activation of astrocytes and microglia, decreased the expression of IL‐1β and the ratio of pNF‐κB/NF‐κB, and activated AMPK and SIRT1 in SOD1^G93A^ mice. ATG upregulates SIRT1 and suppresses IL‐1β expression, and overexpression of SIRT1 has been found to reduce the inflammatory response via suppression of IL‐1β and subsequent NF‐κB.^38^ Therefore, A‐1 treatment exerted an anti‐inflammatory effect in SOD1^G93A^ mice via the AMPK/SIRT1/IL‐1β/NF‐κB pathway.

## 5 CONCLUSION

In conclusion, A‐1 treatment reduced motor neuron loss, improved gastrocnemius atrophy, and further delayed the progression of ALS disease via the AMPK/SIRT1/PGC‐1α pathway, which in turn increased mitochondrial biogenesis, and the AMPK/SIRT1/IL‐1β/NF‐κB pathway, which exerted neuroprotective effects via the reduction of neuroinflammation.

## FUNDING INFORMATION

This study was supported in part by grants from NSFC (82171583), The Key Basic Research Program of Shenzhen Science and Technology Innovation Commission (JCYJ20200109150717745; JCYJ20200109144418639), Shenzhen Key Medical Discipline Construction Fund (SZXK069), and Sanming Project of Medicine in Shenzhen (SZSM201611090).

## CONFLICT OF INTEREST STATEMENT

The authors declare no conflicts of interest.

## Supporting information


Tables S1–S3

Data S1

## Data Availability

The data that support the findings of this study are available from the corresponding author upon reasonable request.
