# Correction to [Arctigenin derivative A‐1 ameliorates motor dysfunction and pathological manifestations in SOD1^G93A^ transgenic mice via the AMPK/SIRT1/PGC‐1α and AMPK/SIRT1/IL‐1β/NF‐κB pathways]

**DOI:** 10.1111/cns.14852

**Published:** 2024-07-08

Xiong B, Yang C, Yang X, et al. Arctigenin derivative A‐1 ameliorates motor dysfunction and pathological manifestations in SOD1G93A transgenic mice via the AMPK/SIRT1/PGC‐1α and AMPK/SIRT1/IL‐1β/NF‐κB pathways. *CNS Neurosci Ther*. 2024;**30**:e14692. doi:10.1111/cns.14692

In the methods section of the “Abstract” section, the text “Muscle atrophy and fibrosis, motor neurons, astrocytes and microglia in the spinal cord were evaluated by H&E, Masson, Sirius Red, Nissl and Immunohistochemistry Staining.” is incorrect. This sentence is an inaccurate description. This should have read “Using immunohistochemistry to assess the activation of astrocytes and microglia, and Nissl staining to evaluate the number of motor neurons in the spinal cord; HE staining and Masson's trichrome staining to assess the degree of atrophy and fibrosis in the gastrocnemius muscle.”

We apologize for this error.

