# Supplementary material for: Arctigenin derivative A‐1 ameliorates motor dysfunction and pathological manifestations in SOD1G93A transgenic mice via the AMPK/SIRT1/PGC‐1α and AMPK/SIRT1/IL‐1β/NF‐κB pathways
Source: CNS Neurosci Ther. 2024 Jun 13;30(6):e14692. doi: 10.1111/cns.14692 (PMC11176200; doi:10.1111/cns.14692)
Supplement: Supplementary file 1 — Tables S1–S3 [file CNS-30-e14692-s002.docx]

**Supplementary tables**

Supplementary Table 1. After a single oral gavage administration of ATG (21.6 mg/kg) in SD rats. The plasma drug concentrations were measured in ng/ml units. n=3.

| **Time (h)** | **201** | **202** | **203** | **Mean** | **SD** |
| --- | --- | --- | --- | --- | --- |
| 0.25 | 2.74 | 4.31 | 13.43 | **6.83** | **5.77** |
| 0.5 | 1.97 | 7.97 | 13.80 | **7.91** | **5.92** |
| 1 | 1.58 | 10.63 | 13.36 | **8.52** | **6.17** |

Supplementary Table 2. After a single oral gavage administration of A-1 (35 mg/kg) in SD rats. The plasma drug concentrations were measured in ng/ml units. n=3.

| **Time (h)** | **A-1** | | | | | **ATG** | | | | |
| --- | --- | --- | --- | --- | --- | --- | --- | --- | --- | --- |
|  | **201** | **202** | **203** | **Mean** | **SD** | **201a** | **202a** | **203a** | **Mean** | **SD** |
| 0.25 | BLQ | BLQ | BLQ | **NA** | **NA** | 5.95083 | 20.4308 | BLQ | **13.19** | **10.24** |
| 0.5 | 1.50538 | BLQ | BLQ | **NA** | **NA** | 16.6284 | 23.4087 | BLQ | **20.02** | **4.79** |
| 1 | BLQ | BLQ | BLQ | **NA** | **NA** | 21.3998 | 27.9299 | BLQ | **24.66** | **4.62** |

Note: 35 mg/kg A-1 and 21.6 mg/kg ATG are equimolar concentrations.

Supplementary Table 3. The dilution ratios and product information of antibodies

| Antibody | Specificity | Type | Dilution | Source | SAT NO. |
| --- | --- | --- | --- | --- | --- |
| p-AMPKα | Phospho- AMPKα(Thr172) | Rabbit | 1/1000 | Cell Signaling | 2535 |
| AMPKα | AMPKα | Rabbit | 1/1000 | Cell Signaling | 2532 |
| Sirt1 | Anti-SIRT1 | Mouse | 1/1000 | Abcom | ab110304 |
| PGC-1α | Anti- PGC1alpha | Rabbit | 1/1000 | Abcom | ab54481 |
| GAPDH | Anti-GAPDH | Rabbit | 1/1000 | Abcom | ab9485 |
| Uqcrfs1 | Anti-UQCRFS1/RISP | Rabbit | 1/1000 | Abcom | ab14746 |
| Cox5a | Anti-COX5A | Mouse | 1/1000 | Abcom | ab180129 |
| ATP5a | Anti-ATP5A | Rabbit | 1/1000 | Abcom | ab14748 |
| Ndufa10 | NDUFA10 | Mouse | 1/1000 | Santa Cruz | sc-376357 |
| SDHB | Anti-SDHB | Mouse | 1/1000 | Abcom | ab14714 |
| p-IκBα | Anti-IKB alpha (phospho S36) | Rabbit | 1/1000 | Abcom | ab133462 |
| IκBα | Anti-IKB alpha | Rabbit | 1/1000 | Abcom | ab32518 |
| pNF-κB | Anti-NF-kB p65 (phospho S536) | Rabbit | 1/1000 | Abcom | ab239882 |
| NF-κB | Anti-NF-κB p65 | Rabbit | 1/1000 | Abcom | ab16502 |
