# Supplementary material for: Arctigenin derivative A‐1 ameliorates motor dysfunction and pathological manifestations in SOD1G93A transgenic mice via the AMPK/SIRT1/PGC‐1α and AMPK/SIRT1/IL‐1β/NF‐κB pathways
Source: CNS Neurosci Ther. 2024 Jun 13;30(6):e14692. doi: 10.1111/cns.14692 (PMC11176200; doi:10.1111/cns.14692)

The Western blot experiment employed the prestained protein marker (catalog number 26616) manufactured by Thermo Fisher Scientific. This reagent references the molecular weight of prestained proteins as depicted in the figure below.

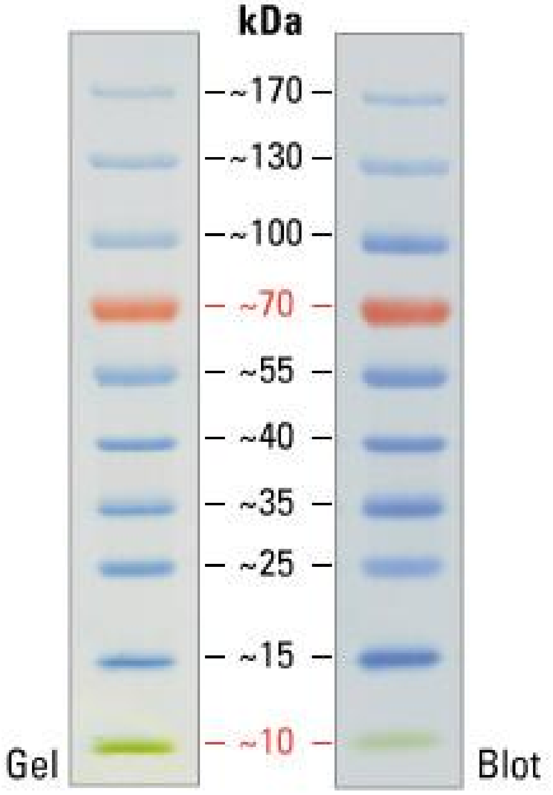

The red dashed box in the figure represents the location of the target protein.

The loading order was: lanes 1-4 for WT group, lanes 5-8 for ALS group, and lanes 9-12 for ALS+A-1 group.

Full unedited blot for Figure 8A (p-AMPKα)

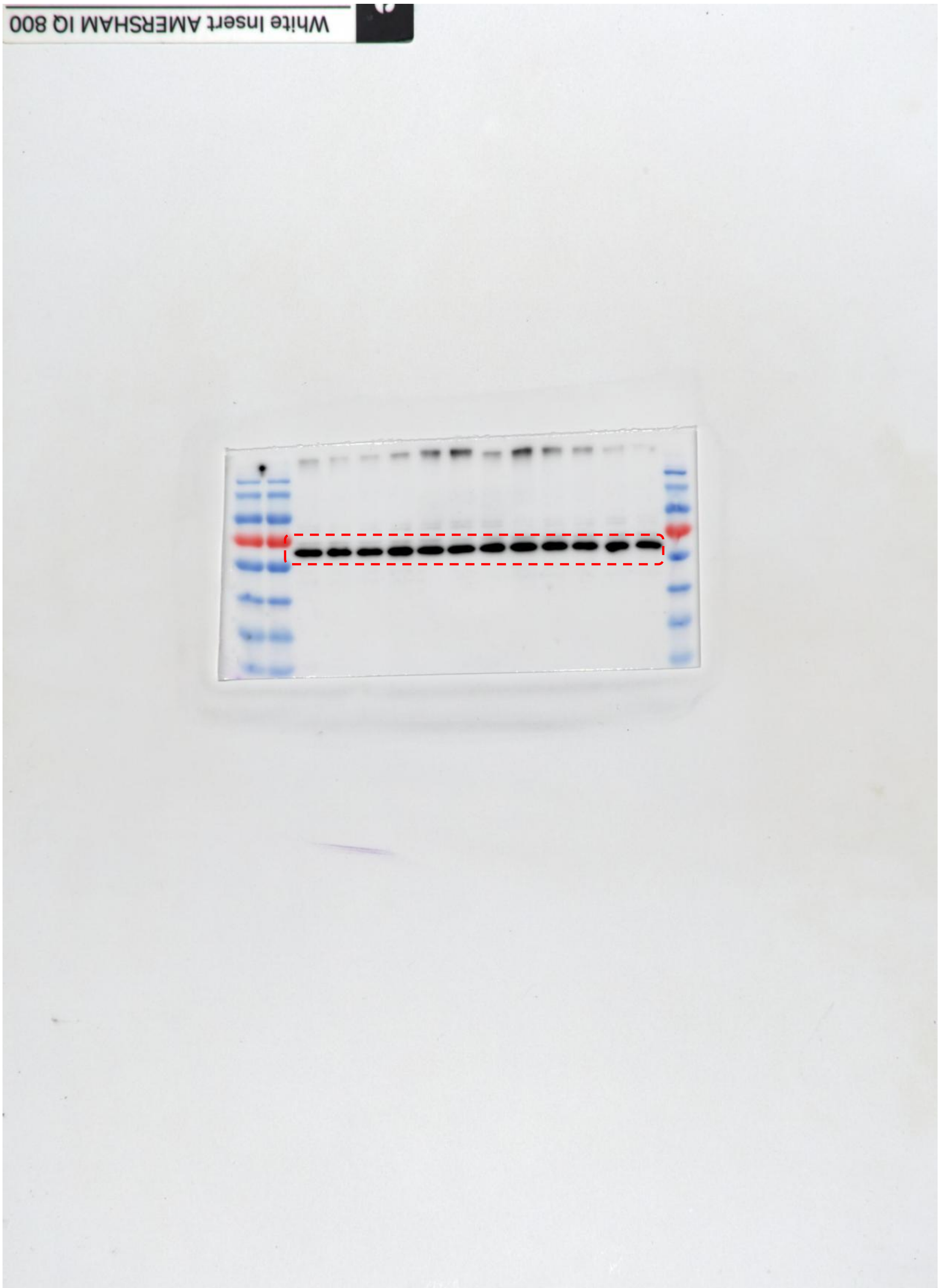

The loading order was: lanes 1-4 for WT group, lanes 5-8 for ALS group, and lanes 9-12 for ALS+A-1 group.

Full unedited blot for Figure 8A (AMPKα)

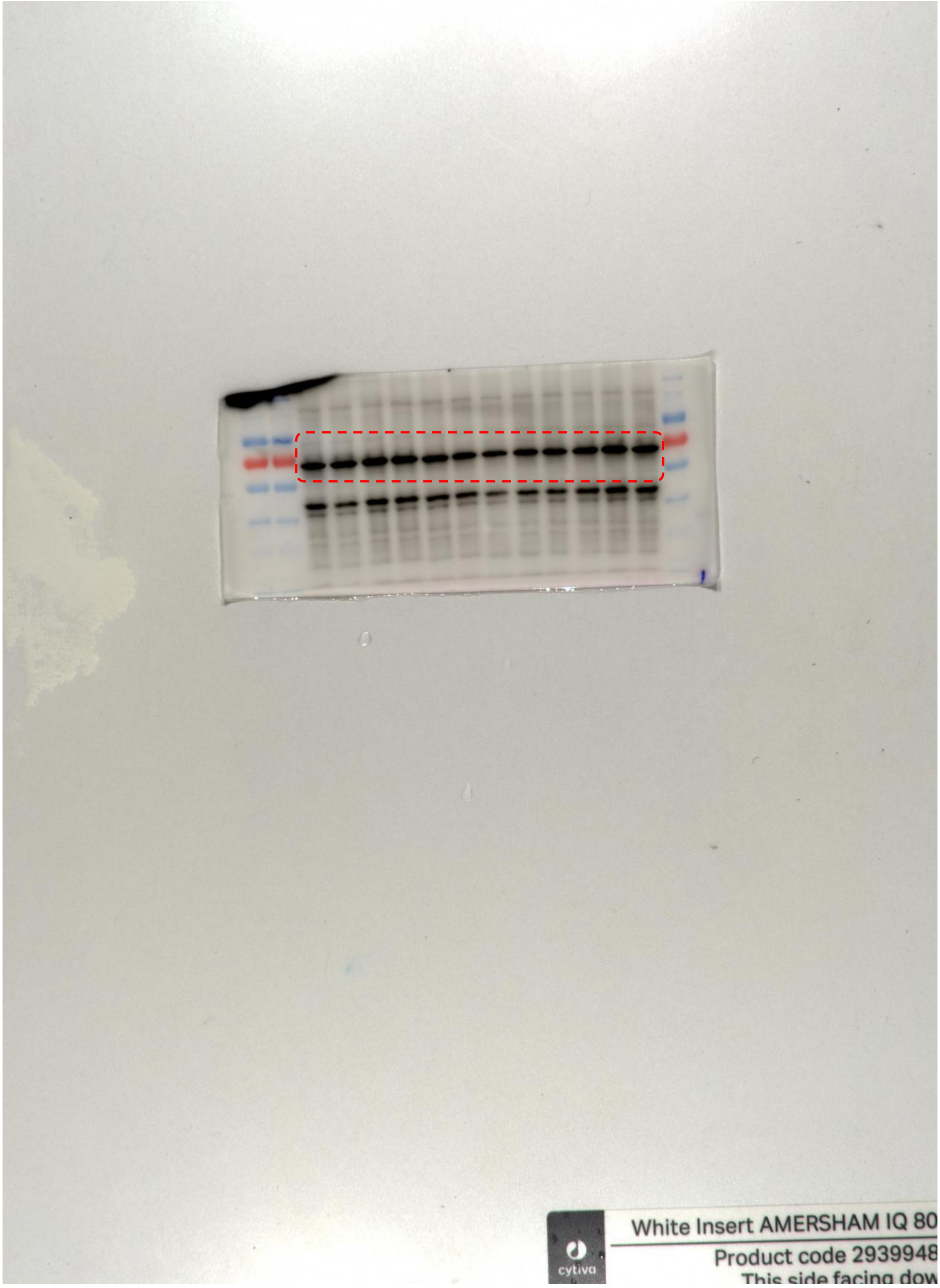

The loading order was: lanes 1-4 for WT group, lanes 5-8 for ALS group, and lanes 9-12 for ALS+A-1 group.

Full unedited blot for Figure 8A (Sirt1)

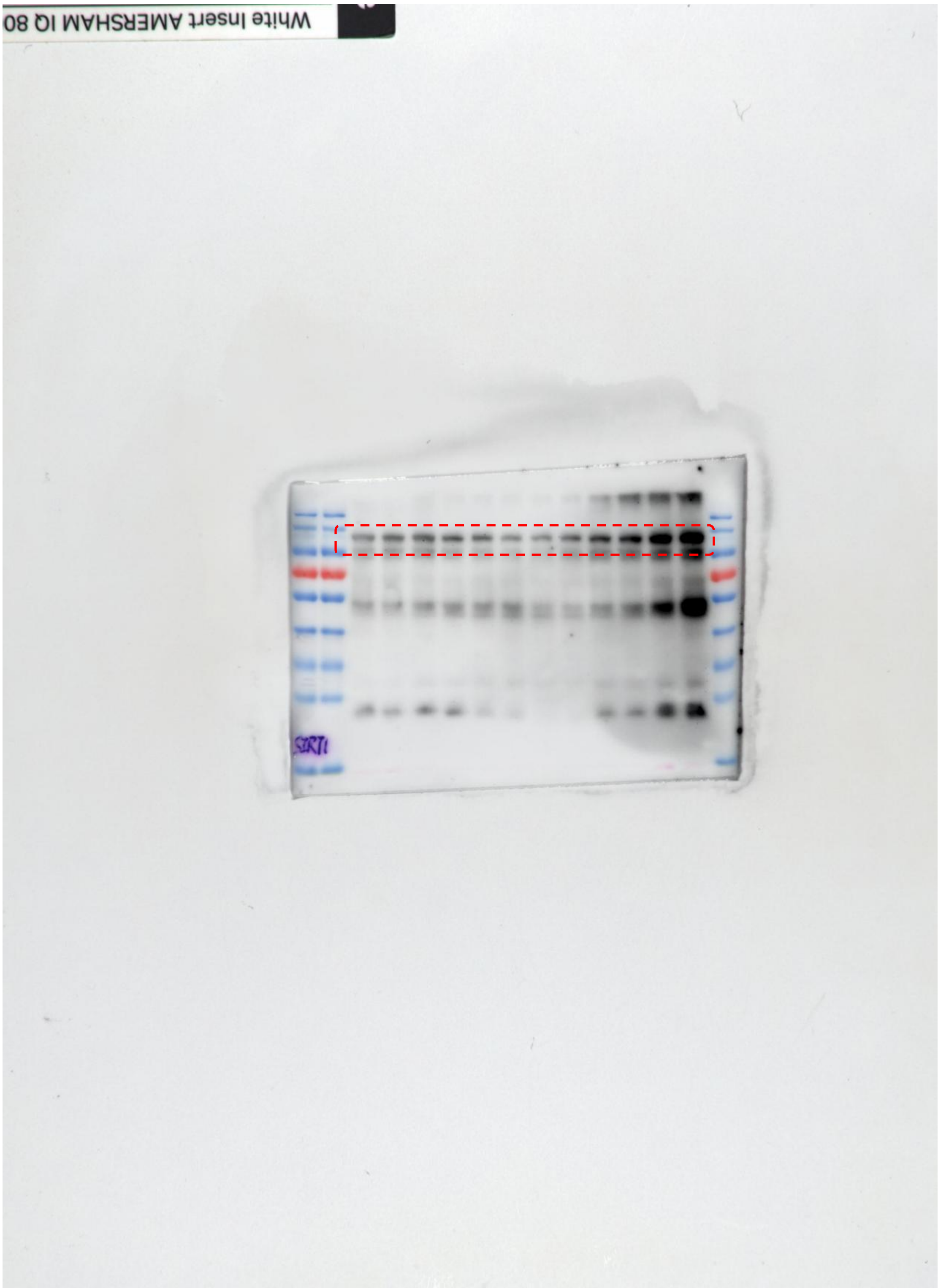

The loading order was: lanes 1-4 for WT group, lanes 5-8 for ALS group, and lanes 9-12 for ALS+A-1 group.

Full unedited blot for Figure 8A (PGC-1α)

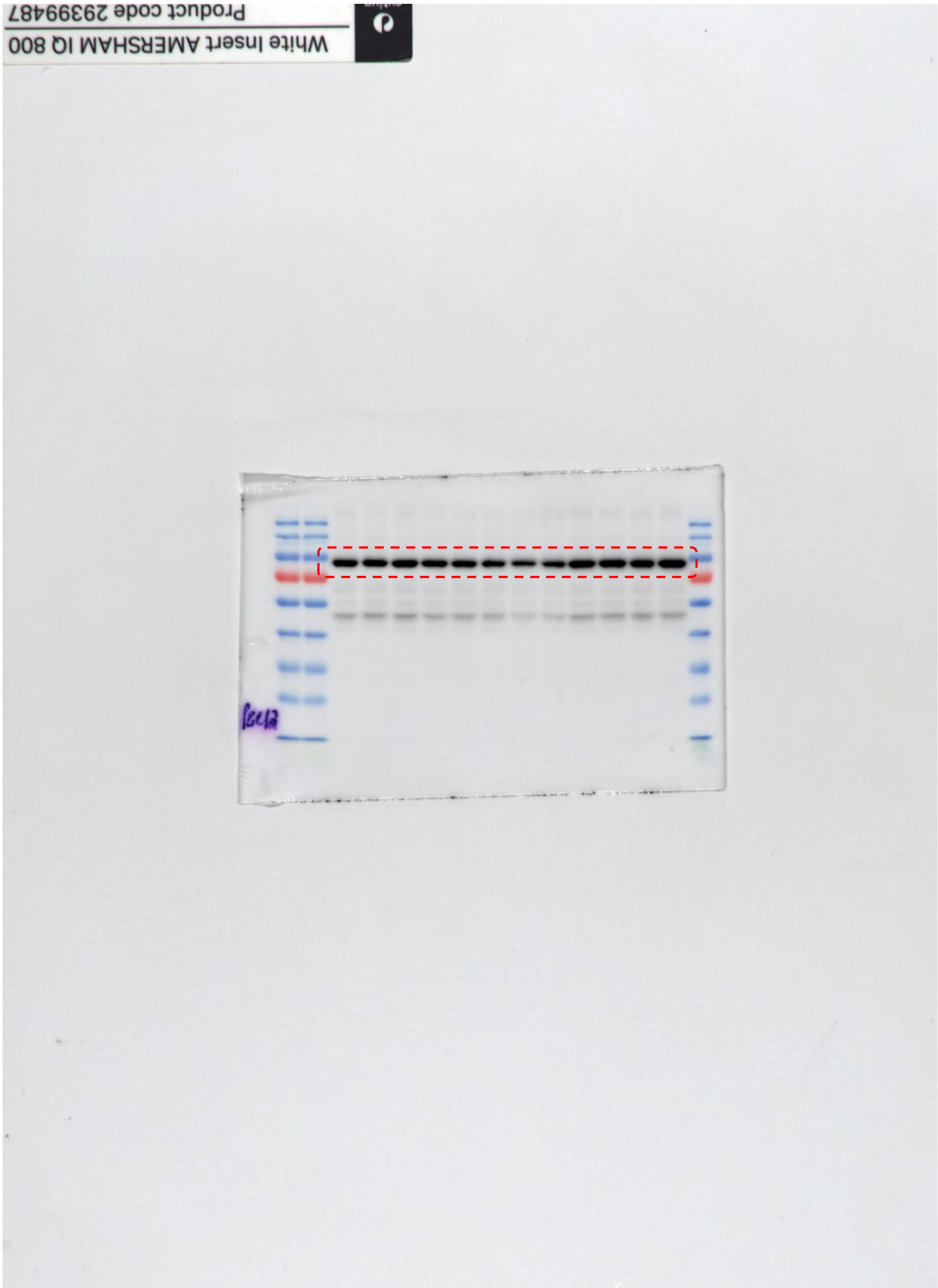

The loading order was: lanes 1-4 for WT group, lanes 5-8 for ALS group, and lanes 9-12 for ALS+A-1 group.

Full unedited blot for Figure 8A (β-actin)

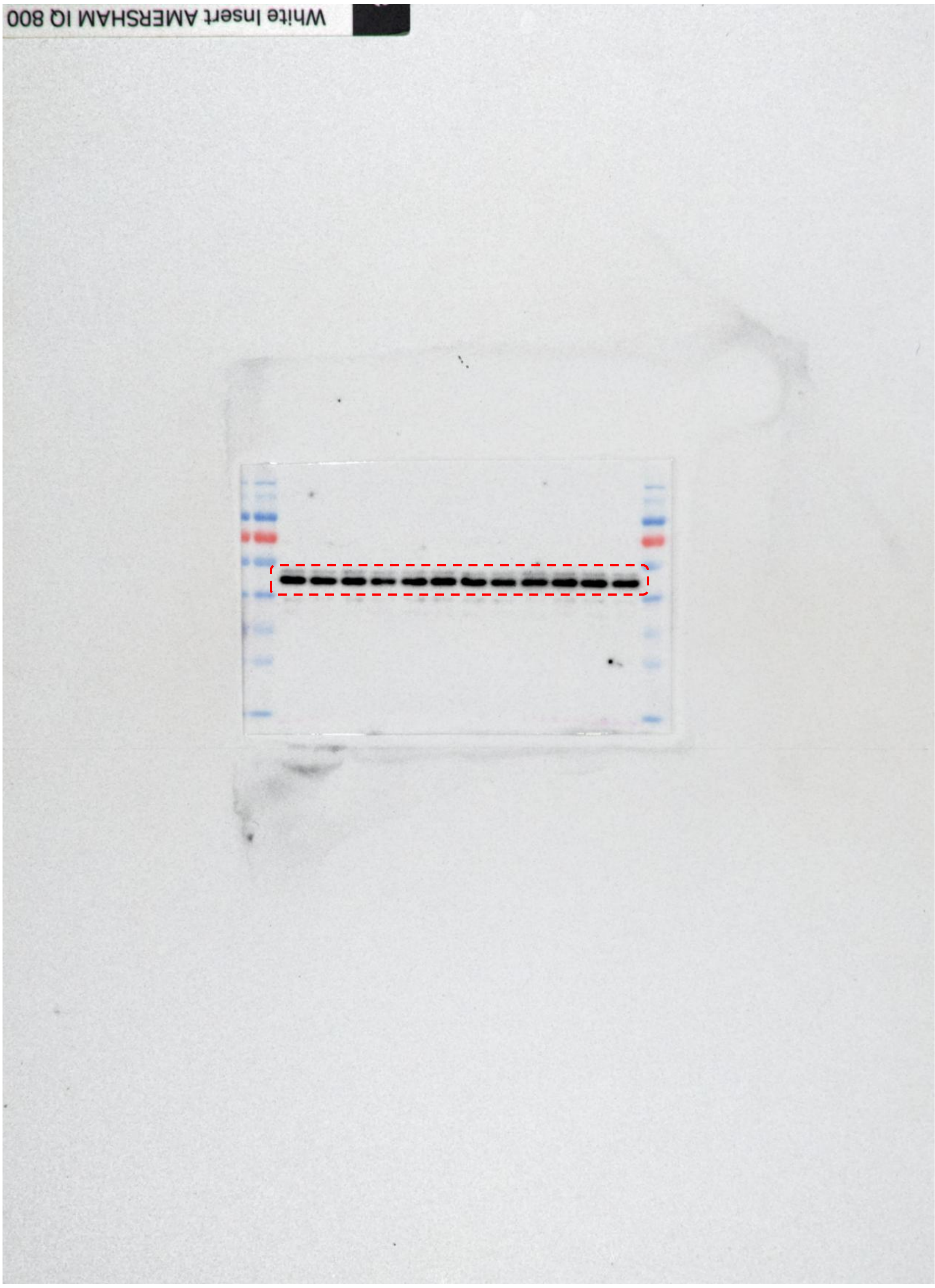

The loading order was: lanes 1-4 for WT group, lanes 5-8 for ALS group, and lanes 9-12 for ALS+A-1 group.

Full unedited blot for Figure 8B (Uqcrfs1)

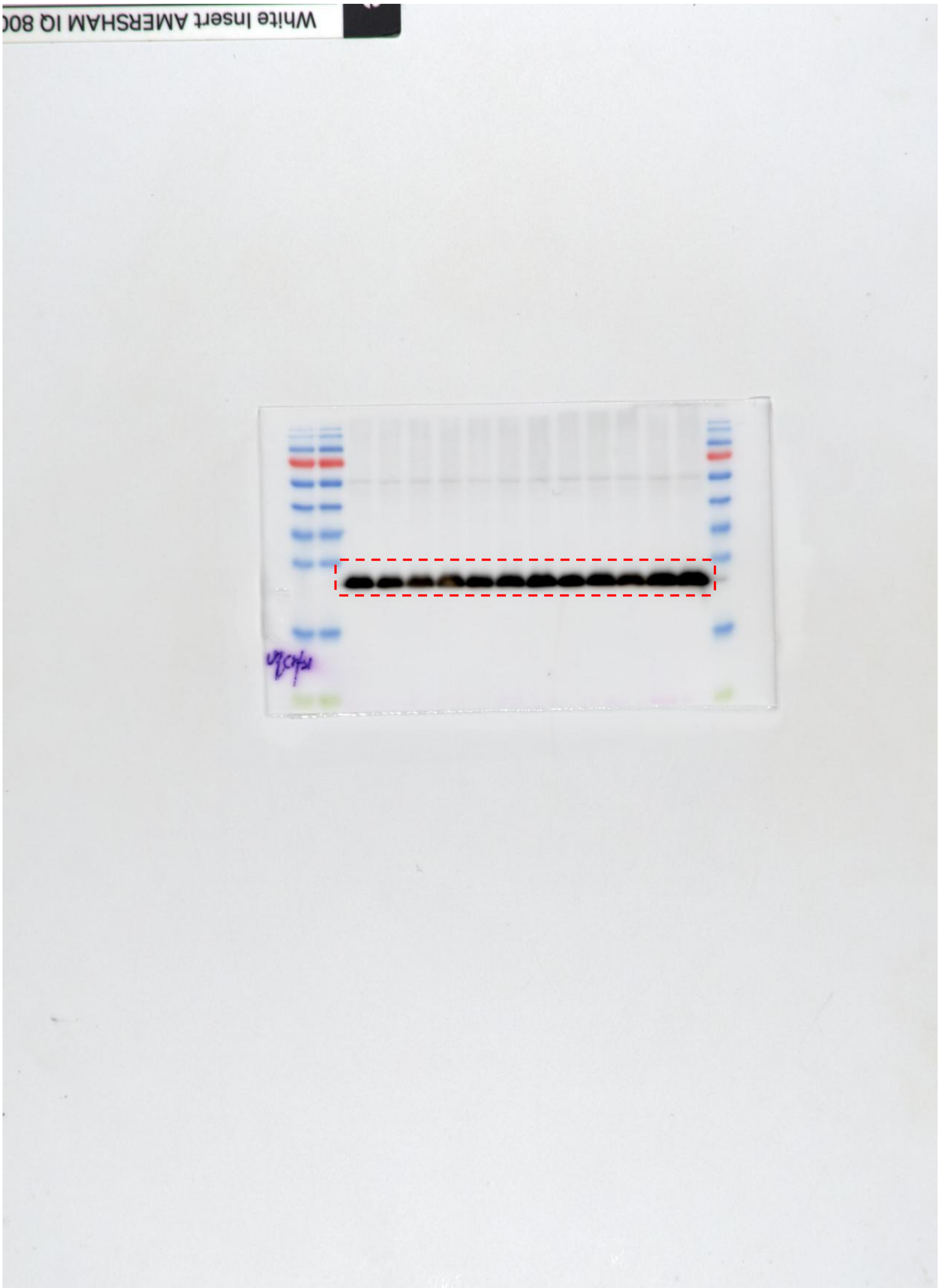

The loading order was: lanes 1-4 for WT group, lanes 5-8 for ALS group, and lanes 9-12 for ALS+A-1 group.

Full unedited blot for Figure 8B (Cox5a)

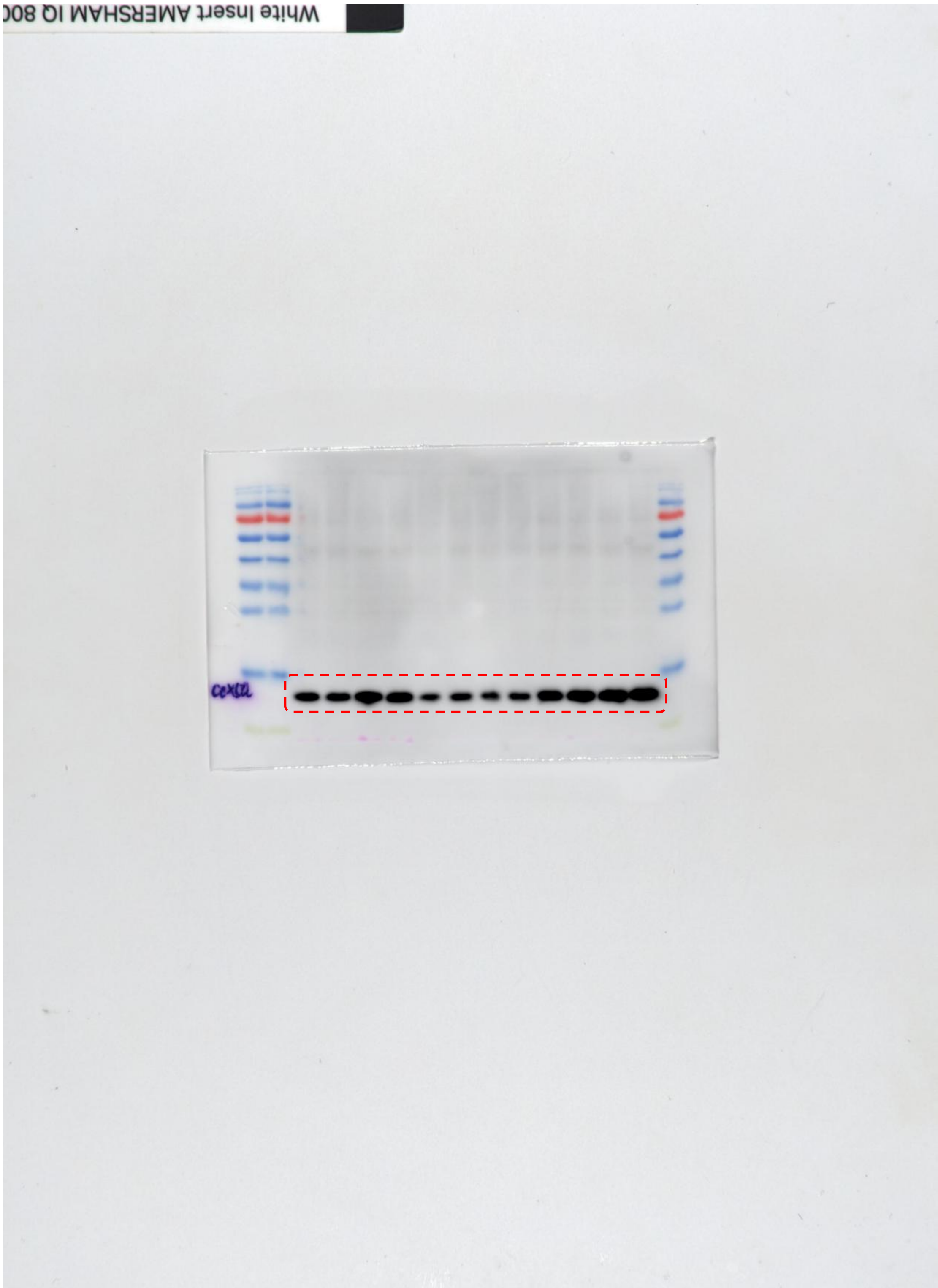

The loading order was: lanes 1-4 for WT group, lanes 5-8 for ALS group, and lanes 9-12 for ALS+A-1 group.

Full unedited blot for Figure 8B (ATP5a)

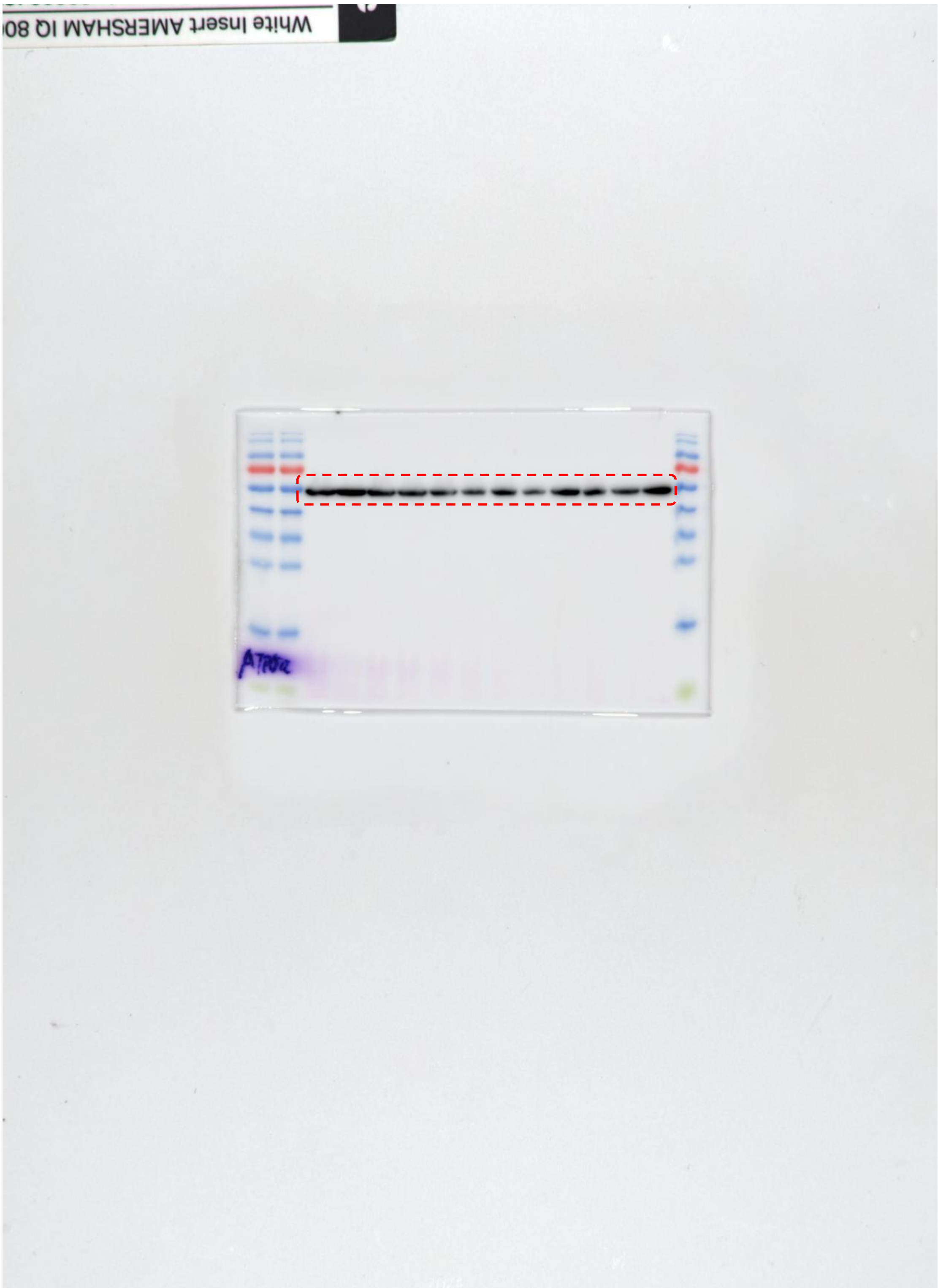

The loading order was: lanes 1-4 for ALS+A-1 group, lanes 5-8 for ALS group, and lanes 9-12 for WT group.

Full unedited blot for Figure 8B (Ndufa10)

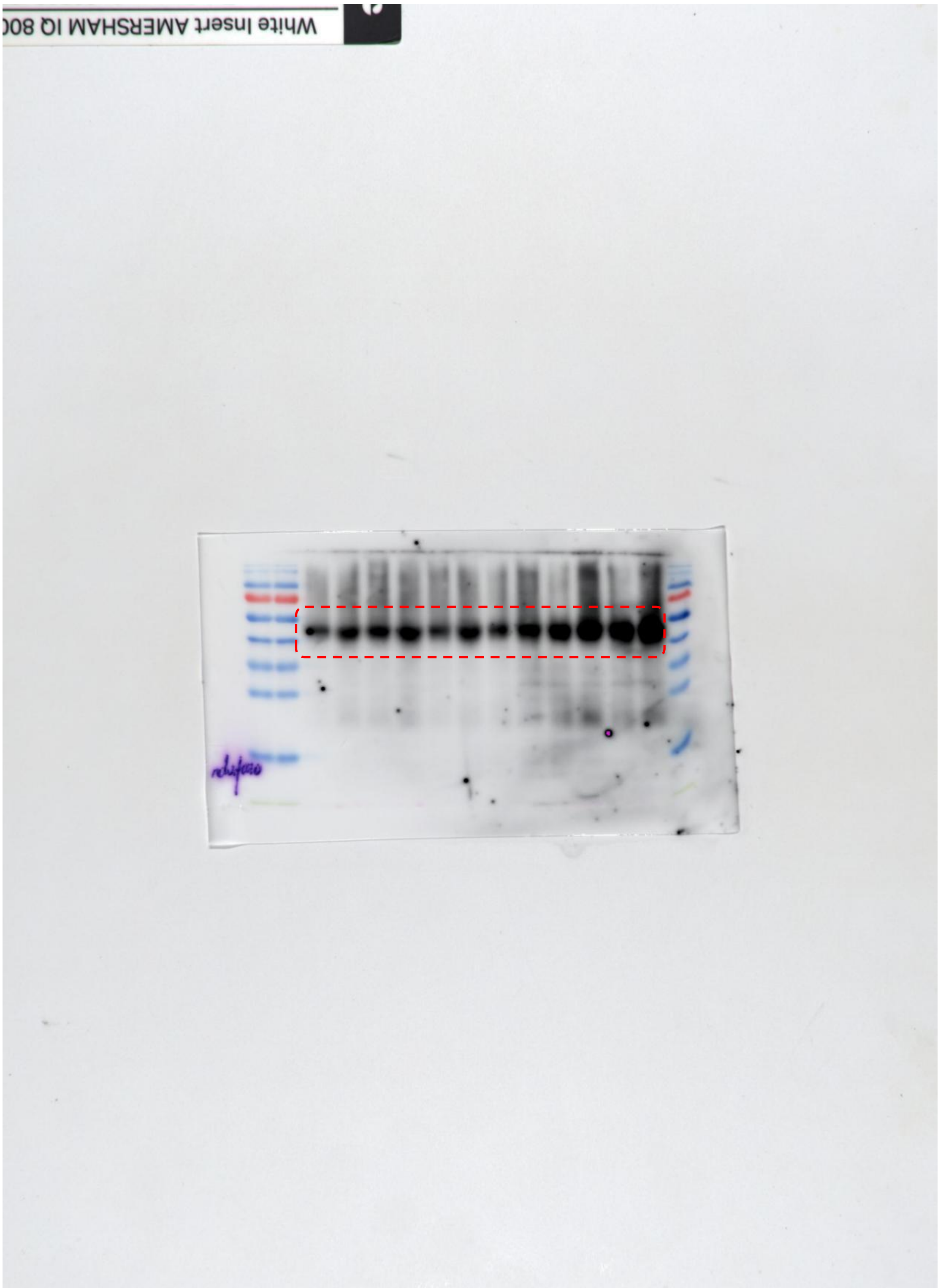

The loading order was: lanes 1-4 for WT group, lanes 5-8 for ALS group, and lanes 9-12 for ALS+A-1 group.

Full unedited blot for Figure 8B (SDHB)

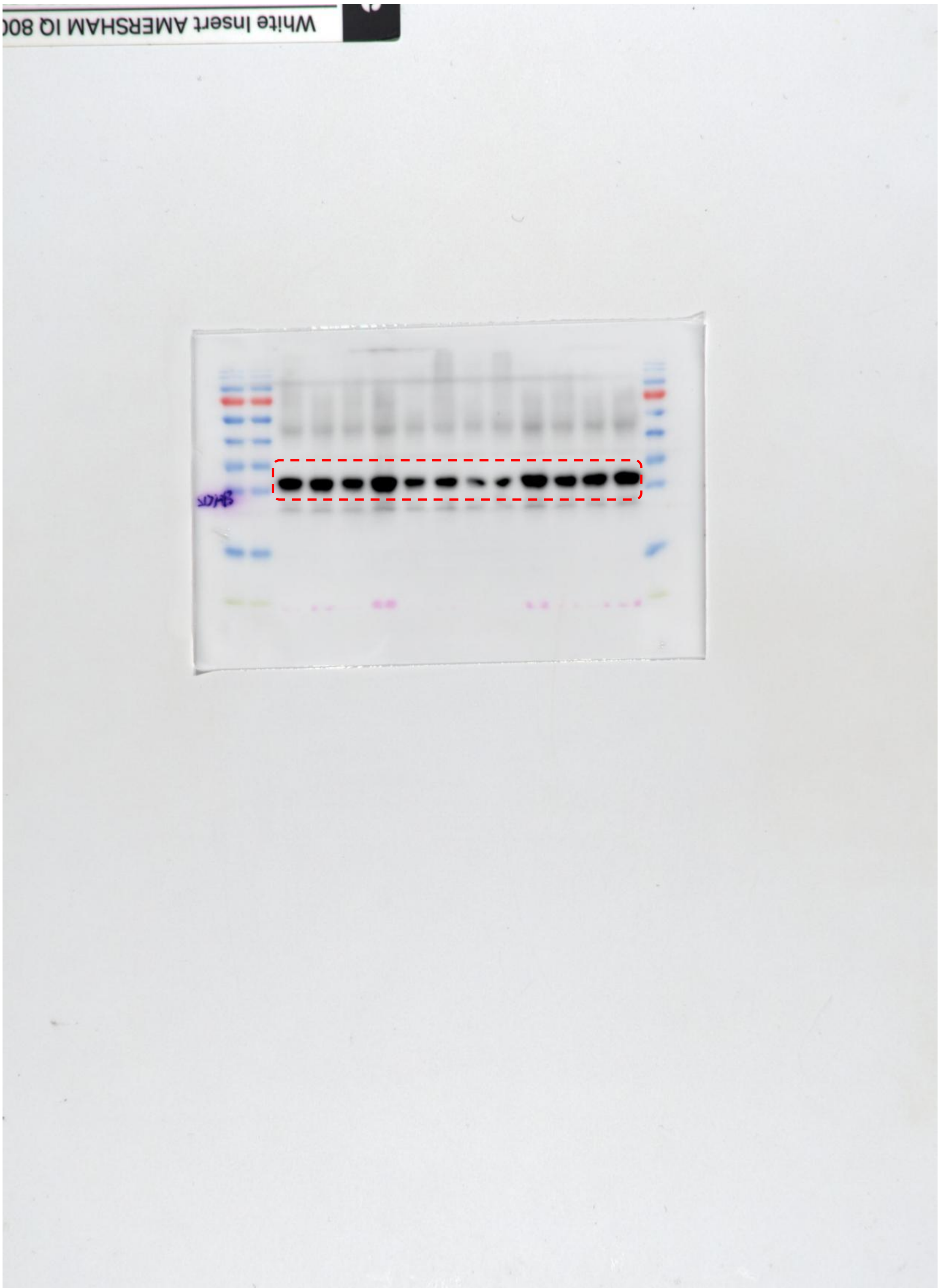

The loading order was: lanes 1-4 for WT group, lanes 5-8 for ALS group, and lanes 9-12 for ALS+A-1 group.

Full unedited blot for Figure 8B (β-actin)

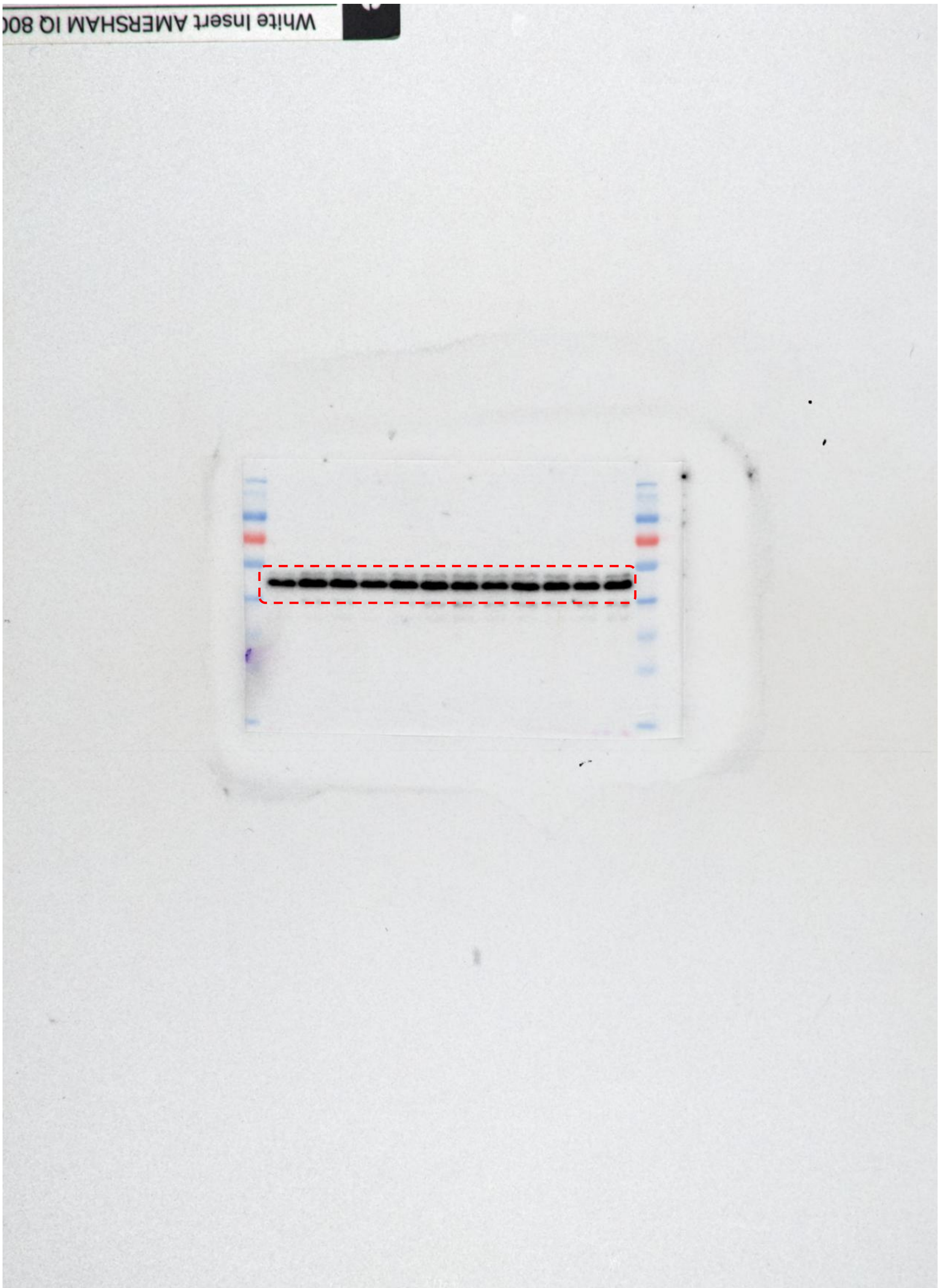

The loading order was: lanes 1-4 for WT group, lanes 5-8 for ALS group, and lanes 9-12 for ALS+A-1 group.

Full unedited blot for Figure 8C (p-IκBα)

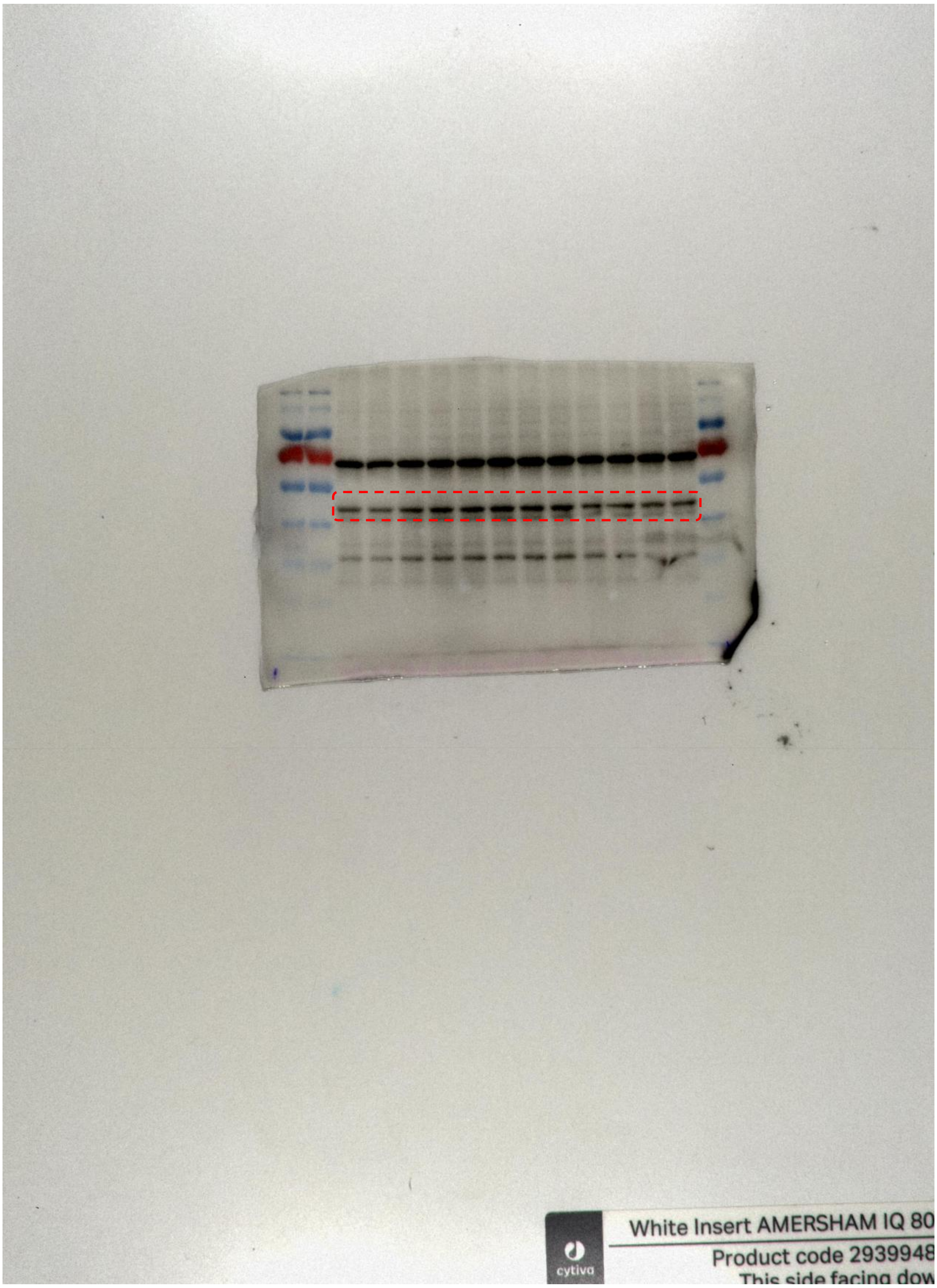

The loading order was: lanes 1-4 for WT group, lanes 5-8 for ALS group, and lanes 9-12 for ALS+A-1 group.

Full unedited blot for Figure 8C (IκBα)

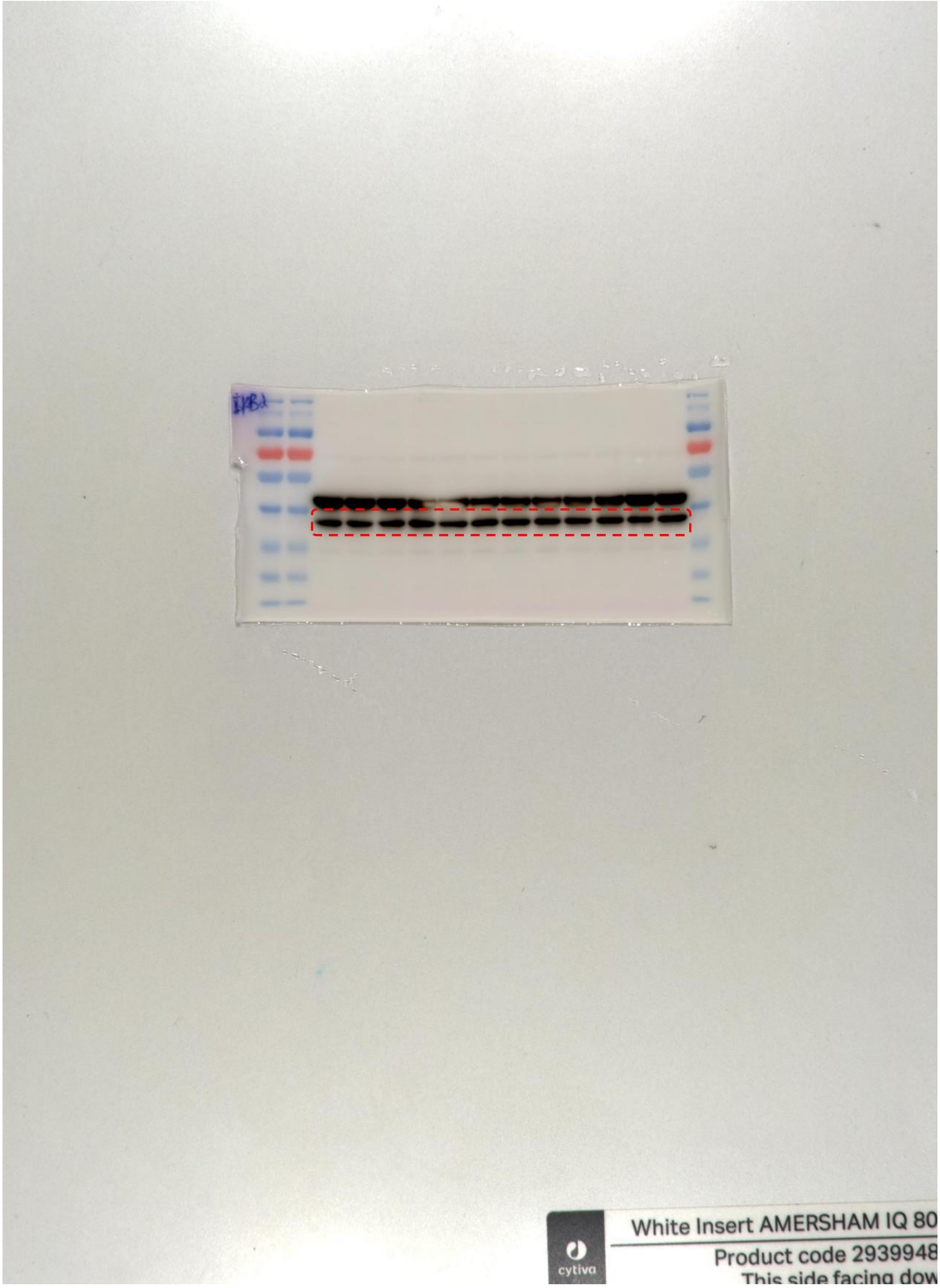

The loading order was: lanes 1-4 for WT group, lanes 5-8 for ALS group, and lanes 9-12 for ALS+A-1 group.

Full unedited blot for Figure 8C (pNF-κB)

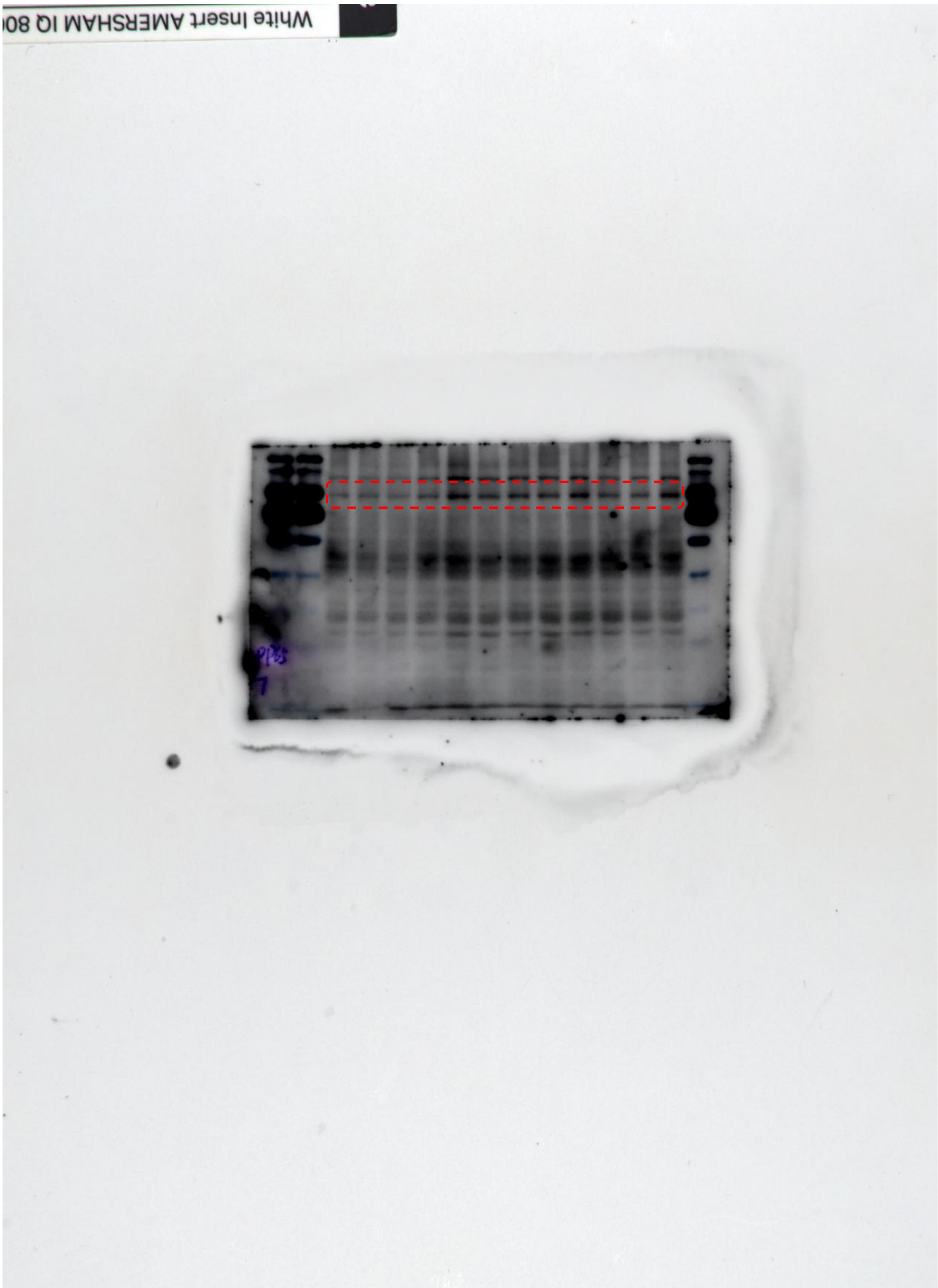

The loading order was: lanes 1-4 for WT group, lanes 5-8 for ALS group, and lanes 9-12 for ALS+A-1 group.

Full unedited blot for Figure 8C (NF-κB)

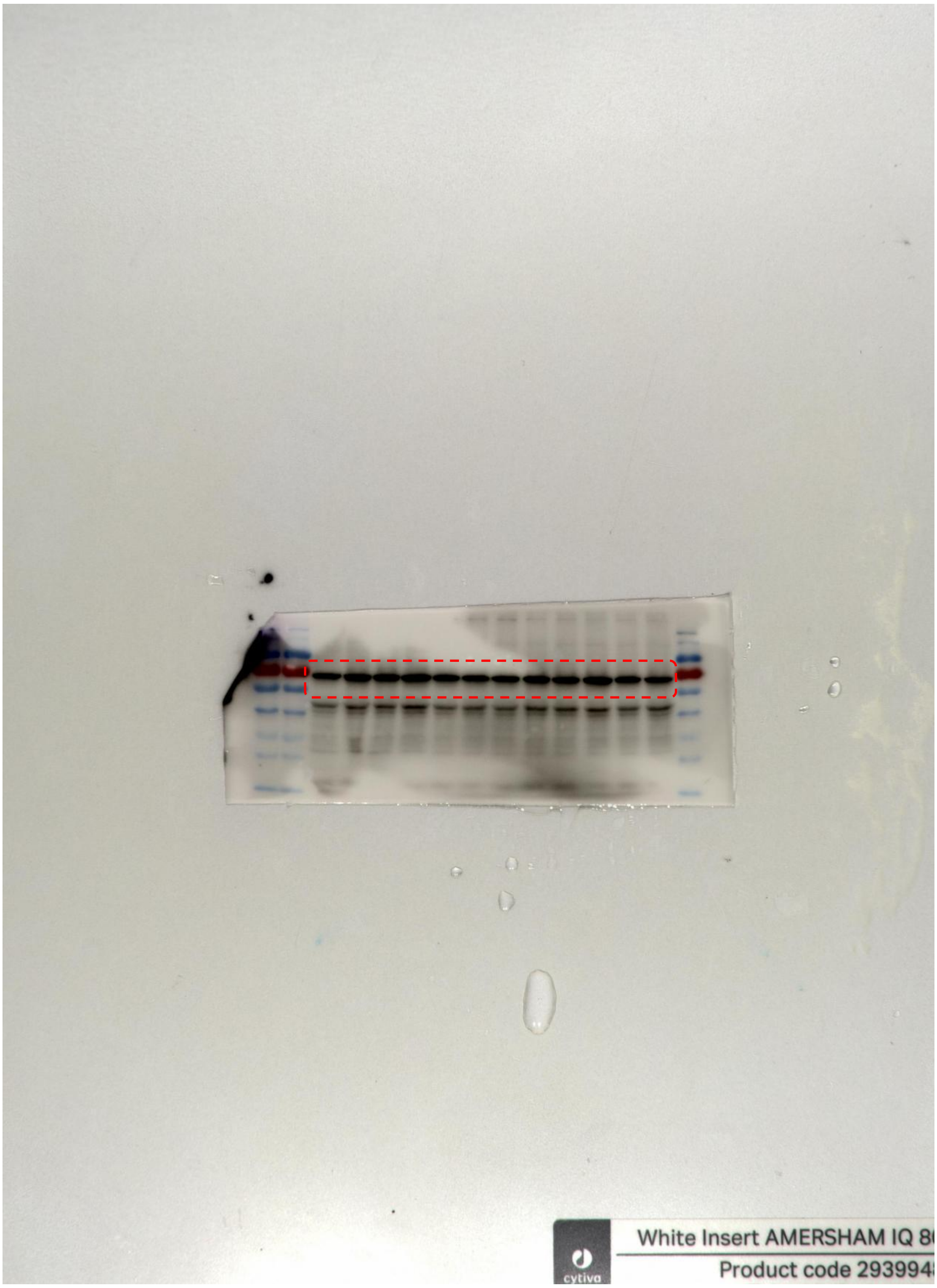

Supplement: Supplementary file 2 — Data S1 [file CNS-30-e14692-s001.pdf]
